# Abstracts of the Cell Therapy Transplant Canada 2023 Annual Conference

**DOI:** 10.3390/curroncol31060223

**Published:** 2024-05-22

**Authors:** Stephanie A. Maier, Imran Ahmad, Tobias Berg, Susan Berrigan, Mahmoud Elsawy, Alejandro Garcia-Horton, Wilson Lam, Alix Lapworth, Luciana Melo Garcia, Ravi M. Shah, Ram Vasudevan Nampoothiri, Jean-Sébastien Delisle

**Affiliations:** 1 Cell Therapy Transplant Canada, Winnipeg, MB R3P 2R8, Canada; 2 Hôpital Maisonneuve-Rosemont, Montreal, QC H1T 2M4, Canada; 3 Faculté de Médecine, Université de Montréal, Montreal, QC H3T 1J4, Canada; 4 Faculty of Health Sciences, McMaster University, Hamilton, ON L8S 4L8, Canada; 5 Hamilton Health Sciences, Hamilton, ON L8N 3Z5, Canada; 6 Alberta Precision Laboratories, Calgary, AB T2N 4Z6, Canada; 7 Queen Elizabeth II Health Sciences Centre, Halifax, NS B3H 3A7, Canada; 8 Faculty of Medicine, Dalhousie University, Halifax, NS B3H 2Y9, Canada; 9 Princess Margaret Cancer Centre, Toronto, ON M5G 2C1, Canada; 10 Faculty of Medicine, CHU de Québec-Université Laval, Québec City, QC G1V 0A6, Canada; 11 Alberta Children’s Hospital, Calgary, AB T3B 6A8, Canada; 12 The Ottawa Hospital, Ottawa, ON K1H 8L6, Canada

**Keywords:** hematopoietic cell transplantation, cell therapy, CAR T-cell therapy, GvHD, lymphoma, leukemia, myeloma, myelodysplastic syndrome

## Abstract

On behalf of Cell Therapy Transplant Canada (CTTC), we are pleased to present the Abstracts of the CTTC 2023 Annual Conference. The conference was held in-person, 31 May–2 June 2023, in Halifax, Nova Scotia at the Westin Nova Scotian hotel. Poster authors presented their work during a lively and engaging welcome reception on Thursday, 1 June, and oral abstract authors were featured during the oral abstract session in the afternoon of Friday, 2 June 2023. Twenty-three (23) abstracts were selected for presentation as posters and four (4) as oral presentations. Abstracts were submitted within four categories: (1) Basic/Translational Sciences, (2) Clinical Trials/Observations, (3) Laboratory/Quality, and (4) Pharmacy/Nursing/Other Transplant Support. The top four (4) oral abstracts and top four (4) poster abstracts were selected to receive an award. All of these were marked as “Award Recipient” within the relevant category. We congratulate all the presenters on their research and contributions to the field.

**Abstract 1 (Poster):** Transcriptomic Characterization of Two Unique Regulatory Natural Killer Cell Subpopulations Associated with Chronic Graft-versus-Host Disease Suppression (**Award Recipient**—Basic and Translational)

Madeline P. Lauener ^1^ (co-first author), Ao Mei ^2^ (co-first author), Sayeh Abdossamadi ^1^, Kirk R. Schultz ^1^, and Subra Malarkannan ^2^

^1^  Michael Cuccione Childhood Cancer Research Program, British Columbia Children’s Hospital Research Institute, University of British Columbia, Vancouver, BC, Canada
^2^  Versiti Blood Research Institute, Division of Hematology and Oncology, Medical College of Wisconsin, Milwaukee, WI, United States of America

**Background:** Chronic graft-versus-host disease (cGvHD) is a major cause of morbidity and mortality after Hematopoietic Stem Cell Transplantation (HSCT). Previously, in large adult and pediatric human cohorts of HSCT patients, we have identified CD56^bright^ regulatory NK (NK_reg_) cells to be associated with the suppression of cGvHD, and cytolytic CD56^dim^ NK cells to be associated with cGvHD development. We hypothesize that unique subpopulations of NK_reg_ cells may be important in the induction of immune tolerance.

**Purpose:** To transcriptionally characterize the unique NK_reg_ cell subpopulations among HSCT patients that associate with cGvHD development or suppression.

**Methodology:** Day 100 pediatric peripheral blood mononuclear cell samples were utilized from the ABLE (PBMTC 1202) trial, including samples from patients that developed cGvHD (*n* = 3) or no cGvHD (*n* = 4). Single-cell RNA sequencing (scRNAseq) was performed on isolated NK cells using the 10× Chromium machine (8000 cells/sample). Single-cell cDNA libraries were sequenced via Illumina Novaseq 6000 to a depth of ~30,000 reads/cell. Raw data from each sample were demultiplexed, aligned to a custom reference genome, and the unique molecular identifier (UMI) counts were quantified using 10× Genomics Cell Ranger software v3.0.0 (default parameters). Single-cell clustering was performed using the Seurat package (v3.0). Statistical analysis was performed using a Wilcoxon Rank-Sum or *t*-test.

**Results:** We investigated for unique NK cell subsets in patients with and without cGvHD. Single-cell transcriptome analysis revealed seven unique NK cell subsets, three of which strongly associate with cGvHD development or suppression. As previously described, we identified a significant cell increase in Cluster 0 (cytotoxic CD56^dim^ NK cells) in patients with cGvHD. Interestingly, we observed two distinct NK_reg_ cell subpopulations associated with cGvHD suppression. We identified an increase in the cell proportions of Cluster 1 (classic CD56^bright^ NK_reg_ cells) and Cluster 2 (Type I interferon-responsive NK_reg_ cells) among immune tolerant patients (Figure 1). Clusters 1 and 2 are characterized by high expressions of the genes *GZMK* and *XCL1* and *IFIT2* and *PMAIP1*, respectively. In both NK_reg_ cell subpopulations, the development of cGvHD was associated with the upregulation of *AREG* and *GZMB* (Table 1).

**Conclusion:** These studies show transcriptionally and quantitatively distinct NK cell subsets associated with cGvHD development, including two unique NK_reg_ cell subpopulations associated with cGvHD suppression. Further analysis may result in a greater understanding of NK_reg_ cell differentiation and the associated mechanisms, which result in immune tolerance.

**Figure 1:**
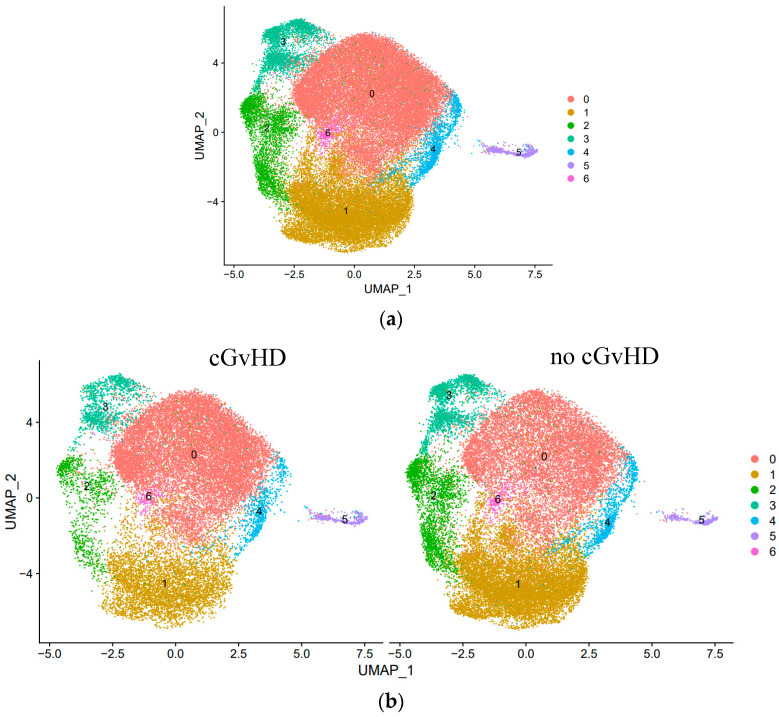
Identification and quantitative analysis of unique NK cell subsets based on single-cell transcriptome among (**a**) all HSCT patients (*n* = 7) and (**b**) HSCT patients that developed cGvHD (*n* = 3) versus no cGvHD (*n* = 4).

**Abstract 2 (Poster):** Measurable Disease and Immune Tumoral Microenvironment in Myeloma Long-Term Survivors After Autologous and Allogeneic Hematopoietic Cell Transplantation

Jean-Sébastien Claveau, Jean-Sébastien Delisle, Jean Roy, Xuehai Wang, Imran Ahmad, Gaël Dulude, Rafik Terra, Gabrielle Boudreau, Guy Sauvageau, and Richard LeBlanc
Institut Universitaire D’Hématologie–Oncologie et Thérapie Cellulaire de Montréal, Hôpital Maisonneuve-Rosemont, University of Montreal, Montreal, QC, Canada

**Background:** Multiple myeloma (MM) remains associated with high morbidity. Only allogeneic (allo) hematopoietic cell transplants (HCTs) may be considered as curative in a minority of patients. In this study, we aimed to define bone marrow (BM) minimal residual disease (MRD) by next-generation flow (NGF) in long-term MM survivors, following either autologous (auto) or allo HCT. Our second aim was to assess the T-cell repertoire.

**Methodology:** In this prospective exploratory study, all the newly diagnosed MM patients with an apparent CR for ≥7 years following auto or tandem auto/allo HCT were eligible. Patients who relapsed were excluded. For mass cytometry (CyTOF) analyses, raw data were normalized, concatenated, and uploaded to the Astrolabe Platform. MRD was assessed with NGF (threshold: 1 tumor cell/10^5^). Next-generation sequencing targeting the hypervariable complementarity-determining region on the T-cell receptor was used to define the T-cell repertoire. The EQ-5D-5L questionnaire was used to assess the quality of life.

**Results:** Between January 2018 and February 2020, 10 and 20 consecutive patients post auto only or tandem auto/allo HCT had a BM examination during their follow-up. Both groups were similar for median age at the time of the auto HCT, immunoglobulin isotypes, and prior therapy. All the patients achieved at least CR at the time of the BM examination. The median follow-up times since HCT were 15 years and 15 years in the auto and auto/allo HCT cohorts, respectively. Only two patients in the allo HCT cohort had a positive MRD versus none in the auto cohort. PFS at 15 years was similar between the auto and allo HCT cohorts (100% vs. 92%, *p* = 0.564). At the time of the median follow-up, 25% of the allo recipients were still taking immunosuppressive therapy. BM assessment of allo recipients was characterized by a significantly increased abundance of the total NKT cells, CD8+ EMRA T-cells, CD4- and CD8- T-cells, and CD4+ memory T-cells compared to the auto cohort. The loss of expression of CD28 and increased expression of CD57 were observed in allo recipients in contrast to auto patients, suggesting T-cell senescence. PD-1 was also increased in the CD4+ Treg population. In contrast, LAG-3, ICOS, OX40, CTLA4, and TIM-3 exhaustion markers were increased in the NKT subpopulation of the auto cohort. The T-cell repertoire was similar between both cohorts. Patients’ EQ-5D-5L showed no difference.

**Conclusion:** We confirmed that long-term PFS is associated with undetectable MRD in most MM patients. We characterized, at a single-cell level, the complexity of the T-cell and NK subset populations in MM survivors following auto and tandem auto/allo HCT. We demonstrated that the late post-allo period is associated with T-cell senescence that could be associated with chronic T-cell stimulation within the BM and perturbed peripheral lymphoid niche homeostasis following alloreactivity and GvHD. The expression of exhaustion markers among NKT cells in auto HCT patients may also suggest similar perturbations.

**Abstract 3 (Poster):** The abstract was withdrawn. The authors were unable to attend and present.

**Abstract 4 (Poster):** Chimeric-Antigen-Receptor-Armed NK Cells Surpass DNAM-1-Mediated Immune Escape Mechanism in Acute Myeloid Leukemia (**Award Recipient**—Clinical Trials/Observations)

Luciana Melo Garcia ^1^, Achintyan Gangadharan ^2^, Gabriel Dominguez ^1^, and Katy Rezvani ^1^

^1^  MD Anderson Cancer Center, Houston, TX, United States of America
^2^  Moffit Cancer Center, Tampa, FL, United States of America

**Background:** NK cells are a part of the innate immune system, and their killing capacity depends on the engagement of activating and inhibitory receptors. DNAM-1 is an essential activating receptor that plays a role in the immune surveillance of acute myeloid leukemia (AML). AML cells develop immune escape mechanisms against NK cell cytotoxicity, including counteracting the DNAM-1-activating potential. Chimeric antigen receptors (CARs) are synthetic receptors integrated into NK cells to direct their anti-leukemia activity. In addition, CD38, a surface protein expressed by AML cells, is a potential target for CAR-based cellular therapy. We hypothesize that CD38-directed CAR NK cells may circumvent DNAM-1-mediated immune escape mechanisms in AML.

**Purpose:** We aimed to investigate the immune escape mechanisms developed by AML cells against the activating activity of DNAM-1 and whether anti-CD38 CAR-armed NK cells can overcome these immune escape mechanisms.

**Methodology:** We studied a sizable single-cell RNA database to determine the DNAM-1 ligand pattern in patients with AML. To characterize the changes in DNAM-1 expression, we co-cultured NK cells with various AML cell lines and performed flow cytometric analysis. We used retrovirally transduced CD38 CAR and CRISPR-Cas9-induced DNAM-1 KO to investigate the impacts of these genetic modifications on NK cell killing against AML.

**Results:** The single-cell RNA study revealed that patients with AML displayed patterns of ligand expression that correlated with clinically meaningful genomic profiles. In addition, the characterization of peripheral blood samples showed that NK cells from patients with AML had significantly lower levels of DNAM-1 than healthy individuals. This lack of DNAM-1 correlated with an aberrant pattern for activating and inhibitory receptors. To confirm the DNAM-1 downregulation in the presence of AML cells, we used NK-AML cell co-culture systems in which we observed a significant downregulation of DNAM-1 that was contact- and CD155-dependent. Furthermore, the CRISPR-Cas9-induced *DNAM-1* knockout (KO) diminished NK cell cytokine production and decreased the killing capacity against AML cells. Using retrovirally CAR-transduced NK cells, CD38-targeting NK cells displayed enhanced and specific cytotoxic activity against primary AML cells. However, when we combined CD38 CAR with *DNAM* KO in NK cells, interestingly, these cells maintained their activating potential, cytokine secretion levels, and killing ability.

**Conclusion:** The lack of DNAM-1 resulted in NK cell dysfunction and dampened activity against AML, which confirmed DNAM-1 as an essential activating receptor. Interestingly, in the context of CD38 CAR-armed NK cells, the lack of DNAM-1 did not alter the anti-leukemic capacity. These findings suggest that CAR-directed NK cells surpass DNAM-1 downregulation as an immune escape mechanism and reinforce the potential of CAR-armed NK cells as a promising alternative for the treatment of patients with AML.

**Abstract 5 (Oral):** 2B4 is a Potent and Targetable Activating Receptor for NK Cell Activation Against Leukemia (**Award Recipient**—Basic and Translational)

Emily B. Carter ^1,2^, Stacey N. Lee ^1,2^, and Jeanette E. Boudreau ^1,2,3^

^1^  Department of Microbiology & Immunology, Dalhousie University, Halifax, NS, Canada
^2^  Beatice Hunter Cancer Research Institute, Halifax, NS, Canada
^3^  Department of Pathology, Dalhousie University, Halifax, NS, Canada

**Background:** Allogeneic hematopoietic stem cell transplantation (HSCT) reveals a potent anti-leukemic role of natural killer (NK) cells, and ongoing clinical trials are transplanting NK cells as adoptive cell therapies. It is understood that the configuration of NK cells with respect to the human leukocyte antigen (HLA) and killer immunoglobulin-like receptor (KIR) impacts transplantation outcomes and that additional germline-encoded activating and inhibitory receptors impact the recognition of cancer cells. So far, however, little attention has been paid to the specific configuration of NK cells given as an adoptive transfer. Interactions between the activating receptor 2B4 and its ligand, CD48, are thought to be important for anti-leukemic activity.

**Purpose:** Strategies to rank, select, and/or engineer NK cells with the greatest alloreactivity may facilitate the use of NK cells as precise immunotherapies for leukemia and other cancers. We hypothesize that cancer immunotherapy could be tailored to the specific receptors available on NK cells and tumor cells if patterns of ligand expression can be predicted on the target cells. We are undertaking a systematic investigation to understand how removing and adding signals impacts NK cell reactivity.

**Methodology:** Peripheral blood NK cells selected from a bank of >400 cryopreserved genotyped healthy human donors were challenged with leukemia cell lines K562, HL-60, or KG-1. Cancer cell and NK cell phenotypes and NK cell activation were assessed using a 26-colour flow cytometry panel or by phosphoflow cytometry to quantitate signal strength in individual NK cells and identify common receptors contributing to NK cell function.

**Results**: Most NK cells express both 2B4 and CD48, a receptor–ligand pair that can bind in cis or trans. When cis 2B4-CD48 interactions on NK cells were interrupted with a 2B4-blocking antibody, NK cell function in both educated and uneducated subsets (trained through NK cell recognition of ‘self’ HLA on healthy cells) was elevated against CD48-negative target cells. Against CD48-positive target cells, however, the addition of the blocking 2B4 antibody had no impact, possibly because the trans presentation was sufficient to break cis 2B4-CD48 interactions on NK cells. Additional simultaneous signals may also impact the NK cell recognition of tumor cells; indeed, the blockade of HLA-KIR interactions enhanced killing against HLA-positive target cells. Ongoing studies will test this by blocking cis interactions with anti-CD48 and blocking additional activating or inhibitory interactions.

**Conclusion:** Our data demonstrate that targeting 2B4 as an activating ligand on NK cells may allow their recruitment for anti-cancer activity but underscore the need to consider multiple receptor–ligand partnerships to predict NK cell function in patients. We expect to continuously refine a hierarchy for activating and inhibitory signals to establish successful and precise NK-cell-based immunotherapies against cancers.

**Abstract 6 (Poster):** Association of Metabolic Tumor Volume (MTV) and Clinical Outcomes in Second-Line (2L) Relapsed/Refractory (R/R) Large B-Cell Lymphoma (LBCL) Following Axicabtagene Ciloleucel (Axi-Cel) Versus Standard-of-Care (SOC) Therapy in ZUMA-7

Frederick L. Locke ^1^, Olalekan O. Oluwole ^2^, John Kuruvilla ^3^, Catherine Thieblemont ^4^, Franck Morschhauser ^5^, Gilles Salles ^6^, Steven P. Rowe ^7^, Saran Vardhanabhuti ^8^, Simone Filosto ^8^, Christina To ^8^, Paul Cheng ^8^, Marco Schupp ^8^, Ronald Korn ^9^, and Marie-José Kersten ^10^

^1^  Moffitt Cancer Center, Tampa, FL, United States of America
^2^  Vanderbilt University Cancer Center, Nashville, TN, United States of America
^3^  Princess Margaret Cancer Centre, University of Toronto, Toronto, ON, Canada
^4^  Department of Hematology–Oncology, Hôpital Saint-Louis, University of Paris, Paris, France
^5^  Centre Hospitalier Universitaire de Lille, University of Lille, Lille, France
^6^  Memorial Sloan Kettering Cancer Center, New York, NY, United States of America
^7^  Johns Hopkins School of Medicine, Baltimore, MD, United States of America
^8^  Kite, a Gilead Company, Santa Monica, CA, United States of America
^9^  Imaging Endpoints, Scottsdale, AZ, United States of America
^10^  Amsterdam University Medical Center, University of Amsterdam, Amsterdam, Netherlands

**Background:** In ZUMA-7 (NCT03391466), axi-cel was superior to the standard of care (SOC) across prognostic subgroups, including tumor burden (TB, calculated by the sum of the product diameters (SPDs)) and lactate dehydrogenase (LDH, Locke et al. ASCO 2022, abstract 7565). TB can also be measured by MTV, which has been shown to correlate with clinical outcomes of chimeric antigen receptor T-cell therapy in third-line R/R LBCL (Hong et al. *Front Oncol*, 2021 and Dean et al. *Blood Adv*, 2020).

**Purpose:** We present clinical outcomes in ZUMA-7 by MTV.

**Methodology:** MTV was obtained from attenuation-corrected whole-body FDG PET scans at screening. Whole-tumor volumes of interest were placed on tumors using a predefined, semiautomated approach. Subsequent radiologist-defined adjustments were applied. MTV was calculated as the number of voxels with standardized uptake value (SUV) measurements between 41 and 100% of the tumor SUV_max_ and reported as MTV_total_ (mL) per patient (pt). Associations were assessed using descriptive statistics.

**Results:** MTV was evaluated in 341 pts (axi-cel: 176, SOC: 165). The median MTV was 228.1 mL (range: 2.3–16,669.3) in axi-cel pts, 231.9 mL (range: 0.04–2811.2) in SOC pts, and 230.2 mL (range: 0.04–16,669.3) overall. The median MTV was higher in pts with elevated (*n* = 185, >median) vs. normal (*n* = 156, ≤median) LDH (371.2 mL [range: 2.3–16,669.3] vs. 123.9 mL [range: 0–3712.8]). MTV was positively associated with SPD (*n* = 308, Spearman correlation: 0.523) and LDH (*n* = 341, Spearman correlation: 0.452). Axi-cel event-free survival (EFS) was superior to SOC for low (≤median, hazard ratio (HR): 0.423) and high (>median, HR: 0.417) MTV. Axi-cel EFS trended shorter in pts with high MTV (HR: 1.441), and EFS was shorter in SOC pts with high MTV (HR: 1.486, Figure 1). The median MTV was higher for axi-cel pts who experienced grades ≥3 neurological events (NEs, *n* = 36) vs. pts who experienced grades 1–2 or no NEs (*n* = 134, 320.9 mL [range: 24.3–13,527.0] vs. 193.9 [range: 2.3–16,669.3]). The median MTV was higher for axi-cel pts who experienced grades ≥3 cytokine release syndrome (CRS, *n* = 11) vs. pts who experienced grades 1–2 or no CRS (*n* = 159, 582.9 mL [range: 114.6–2508.6] vs. 203.3 [range: 2.3–16,669.3]).

**Conclusion:** To the best of our knowledge, this analysis is the first to examine MTV in a large, randomized, prospective R/R LBCL study. Similar to associations of efficacy with SPD and LDH previously observed in 2L LBCL (Locke, FL et al., ASCO 2022, abstract 7565), high MTV was associated with superior outcomes in pts treated with axi-cel vs. SOC. Although the TB per SPD did not seem to impact axi-cel outcomes in ZUMA-7 (Locke, FL et al., ASCO 2022, abstract 7565), high MTV was associated with poorer outcomes with axi-cel vs. low MTV, and rates of grades ≥3 NEs and CRS were associated with higher MTV. This suggests that MTV is a more accurate and sensitive measure of TB vs. SPD. Nevertheless, axi-cel was superior to SOC irrespective of the MTV subgroup, including among pts in the high-MTV subgroup. 

**Figure 1:**
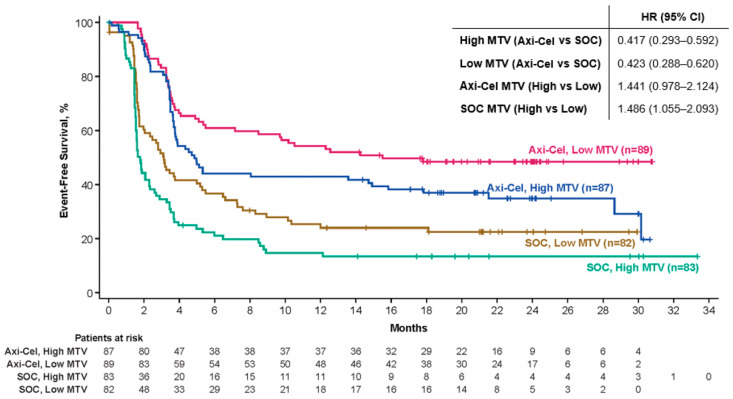
Event-free survival per central review. Figure shows the Kaplan–Meier estimate of EFS by blinded central review in patients with MTV evaluated. EFS was defined as the time from randomization to the earliest date of disease progression, according to the Lugano classification (Cheson et al. *Journal of Clinical Oncology* 2014) of new lymphoma therapy, death from any cause, or the best response of the stable disease up to and including the response of the day-150 assessment after randomization, per blinded central review. Tick marks indicate patients who did not meet the criteria for an event and were censored. Axi-cel, axicabtagene ciloleucel; EFS, event-free survival; HR, hazard ratio; MTV, metabolic tumor volume; SOC, standard of care.

**References** **:**
Cheson BD, Fisher RI, Barrington SF et al. Recommendations for initial evaluation, staging, and response assessment of Hodgkin and non-Hodgkin lymphoma: the Lugano classification. Journal of Clinical Oncology, 32(27):3059–68, 2014.
Dean EA, Mhaskar RS, Lu H et al. High metabolic tumor volume is associated with decreased efficacy of axicabtagene ciloleucel in large B-cell lymphoma. Blood Advances, 4(14):3268–3276, 2020.
Hong R, Tan Su Yin E, Wang L et al. Tumor burden measured by 18F-FDG PET/CT in predicting efficacy and adverse effects of chimeric antigen receptor T-Cell therapy in non-Hodgkin lymphoma. Frontiers in Oncology, 11:713577, 2021.
Locke FL, Chou J, Vardhanabhuti S et al. Association of pretreatment (preTx) tumor characteristics and clinical outcomes following second-line (2L) axicabtagene ciloleucel (axi-cel) versus standard of care (SOC) in patients (pts) with relapsed/refractory (R/R) large B-cell lymphoma (LBCL). Journal of Clinical Oncology, 40:7565–7565, 2022.

**Abstract 7 (Poster):** Validating Alpha-Ketoglutarate as a Biomarker for Chronic Graft-versus-Host Disease in a Separate Pediatric Cohort

Tashi Rastogi ^1^, Bernard Ng ^2^, Liam Johnston ^3^, Sayeh Abdossamadi ^1^, Amina Karimina ^1^, Madeline Lauener ^1^, Elena Ostroumov ^1^, Barnaby Malong ^1^, Dong Jun Zheng ^1^, and Kirk R. Schultz ^1^

^1^  Michael Cuccione Childhood Cancer Research Program, British Columbia Children’s Hospital Research Institute, Vancouver, BC, Canada
^2^  Department of Statistics, Centre for Molecular Medicine and Therapeutics, British Columbia Children’s Hospital, University of British Columbia, Vancouver, BC, Canada
^3^  Department of Pathology and Laboratory Medicine, University of British Columbia, Vancouver, BC, Canada

**Background:** Chronic graft-versus-host disease (cGvHD) is a major long-term complication of allogeneic hematopoietic stem cell transplants (HSCTs) and the most common cause of non-relapse mortality in HSCT patients. Previously, we (Subburaj et al., 2022, Blood) found biological differences associated with cGvHD in a large pediatric study, ABLE1.0/PBMTC1202. In ABLE1.0, patients with moderate–severe cGvHD at disease onset demonstrated significant elevations of α-ketoglutarate, kynurenine, glutamic acid, and 18:0SM, as well as decreases in p-hydroxyhippuric acid, C8, and acetyl-ornithine.

**Purpose:** The objective of this study was to use an independent pediatric cohort to validate the differences in levels of metabolites present in the blood plasma of patients with severe cGvHD using archival samples from a Children’s Oncology Group (COG) therapeutic trial, ASCT0031.

**Methodology:** Seventy-five patients were enrolled in the trial at the onset of severe cGvHD, including 23 patients who did not develop either acute or cGvHD at 3, 6, and 12 months post HSCT. Plasma was separated from whole blood and examined using direct injection mass spectrometry with reverse-phase LC-MS/MS for ~142 metabolites. Differences in metabolite levels between cGvHD (*n* = 38) and non-cGvHD (*n* = 37) patients were compared using multiple regression and considered as statistically significant if a metabolite had (1) a *p*-value of < 0.05; (2) an effect ratio of ≥1.3 or ≤0.75; and (3) a receiver operator characteristic AUC of ≥ 0.60.

**Results:** We found significant increases in α-ketoglutarate and glutamic acid and close to a significant increase in kynurenine at the onset of pediatric cGvHD, as seen in Subburaj et al. We also observed a significant decrease in glutamine. However, an elevation in 18:0SM and decreases in p-hydroxyhippuric acid and acetyl-ornithine were not observed in this data. Curiously, instead of being decreased, C8 was significantly increased in this cohort. See Table 1.

**Conclusion:** This study was able to validate the primary metabolome changes seen previously in a large pediatric cohort, including increases in α-ketoglutarate, glutamic acid, and kynurenine. The significant decrease in glutamine found in this study is consistent with the observed elevation in glutamic acid found in ABLE1.0 and ASCT0031, as these metabolites are usually inversely related. However, secondary metabolome changes, such as an elevation in 18:0SM and decreases in p-hydroxyhippuric acid and acetyl-ornithine, were not found in these data. Contact: tashi.rastogi@bcchr.ca; kschultz@mail.ubc.ca.

**Reference** **:**
Subburaj D, Ng B, Kariminia A, Abdossamadi S et al. Metabolomic identification of α-ketoglutaric acid elevation in pediatric chronic graft-versus-host disease. Blood, 2022 Jan. 13;139(2):287–299.

**Abstract 8 (Poster):** The Effect of the COVID-19 Pandemic on Unrelated Allogeneic Hematopoietic Donor Collections and Safety

Gaganvir Parmar ^1,2^, David S. Allan ^1,2^, Gail Morris ^1^, Nicholas Dibdin ^1^, Kathy Ganz ^1^, Karen Mostert ^1^, Kristjan Paulson ^3,4^, Tanya Petraszko ^1,5^, Nora Stevens ^1^, and Matthew D. Seftel ^1,5^

^1^  Stem Cells Division, Canadian Blood Services, Ottawa, ON, Canada
^2^  Department of Medicine and Biochemistry, Microbiology and Immunology, Faculty of Medicine, University of Ottawa, Ottawa, ON, Canada
^3^  Cell Therapy Transplant Canada, Winnipeg, MB, Canada
^4^  Department of Internal Medicine, Max Rady College of Medicine, University of Manitoba, MB, Canada
^5^  Department of Medicine, Faculty of Medicine, University of British Columbia, Vancouver, BC, Canada

**Background:** The COVID-19 pandemic profoundly influenced unrelated donor (UD) allogeneic peripheral blood stem cell (PBSC) collections. Changes included efforts to minimize COVID-19 exposure to donors and the cryopreservation of products. The extent to which the efficacy and safety of PBSC donations were affected by the pandemic is unknown.

**Methodology:** Prospective cohort analysis of PBSC collections comparing pre-pandemic (1 April 2019–14 March 2020) and pandemic (15 March 2020–31 March 2022) eras.

**Results:** Of a total of 291 PBSC collections, cryopreservation was undertaken in 71.4% of the pandemic donations compared to 1.1% pre-pandemic. The mean requested CD34^+^ cell dose per kilogram increased from 4.9 ± 0.2 × 10^6^ pre-pandemic to 5.4 ± 0.1 × 10^6^ during the pandemic. Despite this increased demand, the proportion of collections that met or exceeded the requested cell dose did not change, and the mean CD34^+^ cell doses collected (8.9 ± 0.5 × 10^6^ pre-pandemic vs. 9.7 ± 0.4 ×10^6^ during the pandemic) remained above requested targets. Central-line placements were more frequent, and severe adverse events in donors increased during the COVID-19 pandemic.

**Conclusion:** The cryopreservation of UD PBSC products increased during the pandemic. In association with this, requested cell doses for PBSC collections increased. Collection targets were met or exceeded at the same frequency, signaling high donor and collection-centre commitment. This was at the expense of increased donor or product-related severe adverse events. We highlight the need for heightened vigilance about donor safety, as demand on donors has increased since the COVID-19 pandemic.

**Abstract 9 (Poster):** A Single-Centre Review of Outcomes of Patients with Relapsed or Refractory Large B-Cell Lymphoma Receiving Chimeric Antigen Receptor T-Cell Therapy: The Nova Scotia Program Experience

Amye M. Harrigan ^1^, Nicholas Forward ^1^, Laura V. Minard ^2^, Christina Fraga ^1^, Darrell White ^1^, Rachel Nielsen ^1^, Yomna Eissa ^3^, K. Sue Robinson ^1^, Sudeep Shivakumar ^1^, Mary-Margaret Keating ^1^, Amy Trottier ^1^, Alfredo Delatorre ^1^, Shannon Murphy ^1^, Erica Kelly ^1^, and Mahmoud Elsawy ^1^

^1^  Department of Medicine, Dalhousie University, Halifax, NS, Canada
^2^  Department of Pharmacy, Nova Scotia Health, Halifax, NS, Canada
^3^  Princess Margaret Hospital, Toronto, ON, Canada

**Background:** Outcomes of patients (pts) with relapsed or refractory (r/r) large B-cell lymphoma (LBCL) are poor. Anti-CD19 chimeric antigen receptor T-cell (CAR T-cell) therapy has emerged as a highly effective treatment option for r/r LBCL. This novel therapy has demonstrated durable responses and a curative potential in these pts. In Canada, CAR T-cell therapy is the standard of care for pts with r/r LBCL who fail two or more lines of systemic therapy. Until recently, eligible patients from Nova Scotia (NS) had to travel to the United States to receive CAR T-cell therapy. In March 2022, the Queen Elizabeth II Health Sciences Centre in Halifax, NS became the first facility in Atlantic Canada to offer CAR T-cell therapy.

**Purpose:** The aim of this study is to identify the characteristics and outcomes of the pts who have received CAR T-cell therapy during the first year of its availability in NS.

**Methodology:** This is a single-centre retrospective study. All the pts who received CAR T-cell therapy between March 2022 and January 2023 were included. Pts’ characteristics and outcomes were obtained by retrospective medical chart review. This analysis is primarily descriptive.

**Results:** As of January 2023, 11 pts with r/r LBCL have received axicabtagene ciloleucel CAR T-cell therapy. All the pts had successful manufacturing, with turnaround times of 20–22 days, and the manufactured T-cells were infused following lymphodepleting conditioning with standard doses of fludarabine and cyclophosphamide. The clinical course and outcomes for nine patients were evaluable at the time of the abstract submission. Baseline pt characteristics are described in Table 1. The median age was 60 years (range: from 23 to 75 years) and 66% (*n* = 6) of the patients were female. Diffuse large B-cell lymphoma was the most common diagnosis (44%, *n* = 4), and pts had received a median of two prior lines of systemic therapy prior to receiving CAR T-cell therapy. The median hematopoietic cell transplant comorbidity index score at the time of the lymphodepletion was 1 (range: 0–3). The most common comorbidity was pulmonarycomplications. The median cytokine release syndrome (CRS) and immune-effector-cell-associated neurotoxicity syndrome (ICANS) grades were 2 (range: 1–3) and 3 (range: 0–4), respectively. All the pts experienced CRS (11% > grade 3), and 88% experienced ICANS (55% > grade 3). There was no treatment-related mortality. At 30 days post CAR T-cell infusion, the overall response rate was 100%, with 55% (*n* = 5) achieving a complete metabolic response, and 44% (*n* = 4) achieving a partial response (Figure 1).

**Conclusion:** In this cohort of real-world pts with r/r LBCL treated with CAR T-cell therapy, we observed high rates of overall responses. It is important to continue to collect and analyze these data for future pts to better understand our centre’s patient population and areas where outcomes can be improved as our local program expands. Further multivariable analysis of the determinants of responses and toxicities are underway as more pts are treated.

**Figure 1:**
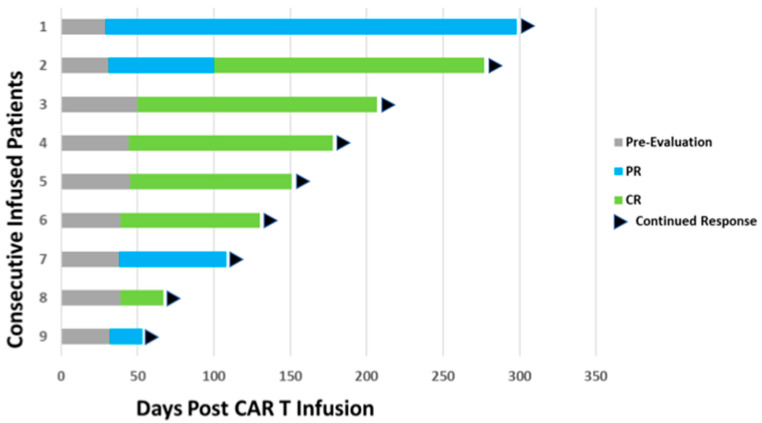
Response rate and duration for consecutive infused patients with relapsed/refractory large B-cell lymphoma treated with axicabtagene ciloleucel in Nova Scotia. PR (partial response) and CR (complete response) per standard PET-CT criteria.

**Abstract 10 (Poster):** Early Experience with Standard-of-Care CAR T-Cell Therapy in Alberta

Robert Puckrin, Ahsan Chaudhry, Krista MacAlister, Judith Olesen, Nizar Bahlis, Jan Storek, Kareem Jamani, Adam Bryant, Jason Tay, Douglas Mahoney, Carolyn Owen, Peter Duggan, Douglas Stewart, and Mona Shafey
Alberta Blood and Marrow Transplant Program, Foothills Medical Centre, Calgary, AB, Canada

**Background:** CAR T-cell therapy has revolutionized the treatment of hematological malignancies but did not become routinely available in Alberta until a provincial CAR T-cell program opened in Calgary in March 2021.

**Purpose:** To evaluate the early experience and clinical outcomes of standard-of-care CAR T-cell therapy in Alberta.

**Methodology:** This study included all the patients ≥18 years old referred for commercial CAR T-cell therapy before November 2022. Progression-free survival (PFS) and overall survival (OS) were determined using the Kaplan–Meier method.

**Results:** Among 41 referred patients, 29 (71%) were approved for treatment, whereas 12 (29%) were ineligible. Two (7%) patients did not proceed to leukapheresis because of rapid disease progression. Among 27 patients who underwent leukapheresis, 22 (81%) received CAR T-cell infusion, whereas five (19%) did not because of disease progression (*n* = 3), fatal infection during bridging therapy (*n* = 1), or manufacturing failure (*n* = 1). Manufacturing failure occurred in three (11%) patients, necessitating a second attempt, which was successful in two cases. The median time from referral to CAR T-cell infusion was 52 days (range: 36–102). Patients from northern Alberta tended to have more frequent delays >30 days from disease indication to referral (35% vs. 12%), greater use of ‘holding chemotherapy’ before leukapheresis (19% vs. 10%), and higher risks for not proceeding to CAR T-cell infusion after approval (29% versus 20%) compared to patients from southern Alberta. However, there was no difference in PFS among infused patients from northern versus southern Alberta (log-rank *p* = 0.88). This infused cohort consisted of 22 patients with a median age of 64 years (range: 22–75) who received axicabtagene ciloleucel (*n* = 14), tisagenlecleucel (*n* = 7), or brexucabtagene autoleucel (*n* = 1) for large B-cell lymphoma (*n* = 20), B-cell acute lymphoblastic leukemia (*n* = 1), or mantle cell lymphoma (*n* = 1). Cytokine release syndrome (CRS) occurred in 18 (82%) patients, including one (5%) patient with grade 3 CRS. Neurotoxicity (ICANS) occurred in seven (32%) patients, including two (9%) patients with grade 3 ICANS. One (5%) patient died of non-relapse mortality 12 months after CAR T-cell infusion because of COVID-19 infection. The overall response rate (ORR) was 86%, with complete responses in 37% and partial responses in 50%. With a median follow-up time of 10 months, the 10-month PFS was 53% and OS was 72% among 22 infused patients (Figure 1B). In an intention-to-treat analysis of 29 patients approved for CAR T-cell therapy, the ORR was 66%, and the 10-month PFS was 40% and OS was 56% (Figure 1A).

**Conclusion:** Standard-of-care CAR T-cell therapy in Alberta has a manageable safety profile and favourable efficacy, which mirrors the results of clinical trials and other real-world studies. Further work is needed to optimize the geographic distance to treatment centres and to reduce the risk of drop-out prior to infusion, which represent significant barriers to the successful delivery of CAR T-cell therapy.

**Figure 1:**
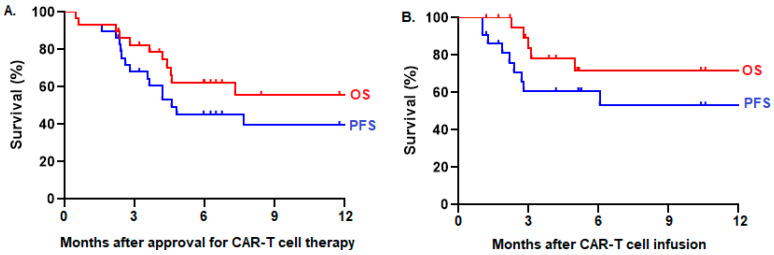
PFS and OS of patients who were (**A**) intended for CAR T-cell therapy (*n* = 29) and (**B**) who received CAR T-cell infusion (*n* = 22) in Alberta.

**Abstract 11 (Poster):** Outcomes of Tandem Autologous Hematopoietic Stem Cell Transplants in High-Risk Myeloma Patients: A Single-Centre Experience

Shona Philip, Selay Lam, Chai Phua, Martha Louzada, Anargyros Xenocostas, and Uday Deotare
Department of Medicine, Division of Hematology, Schulich School of Medicine & Dentistry, Western University, London, ON, Canada

**Background:** Multiple myeloma (MM) is a clinical and genetically complex heterogeneous disease. Patients with high-risk features include at least one of the prognostic factors at diagnosis: t (4;14), t (14;16), t (14;20), del17p, gain 1q amp, and 1q + del1p, which are associated with a poor prognosis in MM. Autologous stem cell transplantation (ASCT) and the development of new agents have considerably increased the median survival of MM patients. Patients with high-risk cytogenetics are associated with worse survival, and studies have shown improvement in progression-free survival with tandem ASCT.

**Methodology:** This is a retrospective single-centre study, where we have described the demographic and clinical characteristics of MM patients with high-risk cytogenetics at our centre who underwent a tandem autologous transplant (Auto–Auto) from 1 January 2017 to 1 January 2021. Secondary objectives looked at progression-free survival (PFS) and the overall survival (OS) using the Kaplan–Meier method.

**Results:** From 1 January 2017 to 31 December 2020, there were 25 high-risk patients who underwent tandem ASCT. Key patient characteristics are shown in Table 1. Translocation (4:14) was seen in 8/25 (32%) patients, t (14:16) in 5/25 (20%) patients, del17p in 7/25 (28%) patients, +1qamp in 8/25 (32%) patients, del1p + 1qamp in 5/25 (20%) patients, and more than one abnormality in 12/25 (48%) patients. In this high-risk group, the most common induction regimen consisted of cyclophosphamide, bortezomib, and steroids (CyBorD) in all the patients, with two patients transitioned to daratumumab, lenalidomide, and dexamethasone and PAD/CVD because of poor responses. Maintenance therapy was given to 19/25 (76%) post-tandem ASCT patients. In terms of responses, we recorded CR (complete response) vs. VGPR (very good partial response) vs. PR (partial response) after induction, 2–3 months after tandem #1, 2–3 months after tandem #2, 12 months after tandem #2, and 12 months after maintenance. Following induction, 16 patients had achieved VGPR, and nine patients had achieved PR. After the first tandem ASCT, five of the previous PR patients had achieved VGPR. In addition, 1–3 months post tandem #2 ASCT, three of the previous PR patients achieved VGPR, and two of the VGPR patients achieved CR. In addition, 12 months after tandem #2, two of the previous VGPR patients achieved CR, and one PR patient achieved VGPR. At the time of the median follow-up, 44% of the patients had relapsed, with a median of 60 months. One patient relapsed within 1–3 months post tandem #1 ASCT. Nine other patients relapsed post tandem #2 ASCT and during maintenance, except for one patient, who relapsed at the time of the tandem #2 ASCT. The 3-year PFS for patients who received tandem ASCT followed by maintenance was not reached (*p* = 0.0001).

**Conclusion:** In our retrospective study, the results suggest that in high-risk MM patients, tandem ASCT allows for achieving deeper responses followed by maintenance therapy to maintain durability and contribute to further PFS duration.

**Abstract 12 (Oral):** A Real-World Analysis of CAR T-Cell Cancellations from a Canadian Cell Therapy Referral Centre (**Award Recipient**—Clinical Trials/Observations)

Eshrak Al-Shaibani ^1^, Eshetu Atenafu ^2^, Rhida Bautista ^1^, Andrew Winter ^1^, Anca Prica ^1^, Michael Crump ^1^, John Kuruvilla ^1^, Vishal Kukreti, ^1^ Robert Kridel ^1^, Arjun Law ^1^, Ivan Pasic ^1^, Sam Saibil ^1^, Wilson Lam ^1^, Sita Bhella ^1^, and Christine Chen ^1^

^1^  Division of Medical Oncology and Hematology, Princess Margaret Cancer Centre, Toronto, ON, Canada
^2^  Department of Biostatistics, Princess Margaret Cancer Centre, Toronto, ON, Canada

**Background:** CAR T-cell therapy (CAR-T) is a potentially curative treatment for aggressive large-cell lymphoma. CAR-T success in the real world can be constrained by restricted resources, systemic delays, and manufacturing inefficiencies, and with few CAR-T centres in Canada, the need to provide services across regional borders (out of province; OOP). We reviewed patient data from the time ofreferral, initial assessment, cell collection, and infusion, identifying causes for patient attrition.

**Methodology:** We performed our first commercial CAR T-cell infusion in June 2020. At that time, there were only three CAR-T centres in Canada using two CD19 CAR-T products: axicabtagene ciloleucel (axi-cel) and tisagenlecleucel (tisa-cel). A chi-squared/Fisher test was used for categorical covariates and a Kruskal–Wallis test for continuous variables, comparing between patients receiving and not receiving CAR T-cell infusion.

**Results:** We analyzed 101 patients referred for CAR T-cell therapy from June 2020 to December 2021; 28 (28%) from OOP. The median age was 61 years (22–81); lymphoma histologies included DLBCL (51%), high-grade lymphoma (21%), transformed lymphoma (18%), PMBCL (5%), and other/missing (6%). Twenty-four percent of the patients had double/triple-hit lymphoma; 54%, refractory disease. Of the 101 patients, 33 (33%) did not reach CAR T-cell infusion. A total of 4/33 patients (12%) were declined at triage because of poor ECOG, CNS disease, unconfirmed relapse histology, or alternative therapy favoured. Of the remaining 29 patients who underwent the initial assessment, 12 (41%) did not proceed to T-cell collection due to: patient decline (*n* = 4), disease progression (*n* = 5, including two with active CNS disease), severe comorbidity (*n* = 1), and alternative therapy chosen (*n* = 2). Of the 17 patients who proceeded to T-cell collection, reasons for not proceeding included failed manufacturing (*n* = 8; seven tisa-cel and one axi-cel), disease progression (*n* = 8, four with active CNS disease), and one declined. The eight patients unable to reach cell infusion because of disease progression had high-risk features at relapse (all stage IV, IPI score > 2, and high LDH), 50% double-hit disease, 75% refractory, and 50% CNS disease. To identify a priori predictors for cancellation amongst collected patients, we compared patients who proceeded to CAR T-infusion to those who did not (Table 1). Aggressive disease features, such as CNS involvement (active or previous) and double/triple-hit status, predicted failure to proceed to infusion. Lower absolute lymphocyte counts and elevated inflammatory markers at apheresis were increased in cancelled patients.

**Conclusion:** We report that one-third of the referrals did not receive CAR T-cell infusion. Half of these cancelled before cell collection, many because of aggressive disease progression and active CNS disease. CNS disease was also a primary reason for cancellation after collection, emphasizing the unmet need for improved CNS disease control through the CAR T-cell therapy process. Cancellations did not appear because of delays in timeline metrics for OOP referrals.

**Abstract 13 (Oral):** HLA-Haplotype Redundancy and Rareness in Canadian Blood Services’ Stem Cell Registry and Cord Blood Bank: Novel Metrics for Optimizing Donor Usage? (**Award Recipient**—Clinical Trials/Observations)

Adrian J. M. Bailey ^1,2^, John Blake ^2,3^, Kathy Ganz ^2^, Matthew Seftel ^2,4^, and David S. Allan ^2,5,6^

^1^  Faculty of Medicine, McGill University, Montreal, QC, Canada
^2^  Canadian Blood Services, Stem Cells and Centre for Innovation, Ottawa, ON, Canada
^3^  Department of Industrial Engineering, Dalhousie University, Halifax, NS, Canada
^4^  Faculty of Medicine, University of British Columbia, Vancouver, BC, Canada
^5^  Clinical Epidemiology and Regenerative Medicine, Ottawa Hospital Research Institute, Ottawa, ON, Canada
^6^  Faculty of Medicine, University of Ottawa, Ottawa, ON, Canada

**Background:** Many patients needing allogeneic hematopoietic cell transplantation (HCT) cannot find HLA-matched donors, especially within some ethnic groups. Understanding the extent of redundancy and rareness of HLA phenotypes in donor inventories may allow strategic choices for optimizing donor recruitment and allow the creation of subinventories for new applications that do not require HLA matching.

**Methodology:** HLA phenotype rareness was determined by matching a donor’s HLA profile against known HLA haplotype frequencies, and redundancy was determined through pairwise comparison of all the donor profiles within an inventory.

**Results:** Among 444,611 total registrants in the CBS’ Stem Cell Registry (SCR), 61,730 registrants were typed at high resolution at five HLA loci (56.9% Caucasian (CAU), 27.1% Asian-Pacific Islander (API), 2.2% African American/Black (AFA), 1.8% North American Indian/First Nations or Metis (NAM), 1.3% Hispanic (HIS), and 10.7% other (OTH)). Overall, 6.6% of the HLA phenotypes in the CBS’ SCR are redundant with variations across ethnic groups (8.3% of CAU, 6.8% of NAM, 4.4% of API, 2.1% of HIS, 0.7% of AFA, and 4.5% of OTH). The total number of registrants with redundant HLA phenotypes was 18.5% (23.9% of CAU, 18.1% of NAM, 10.3% of API, 5.9% of HIS, 1.7% of AFA, and 12.0% of OTH donors). All 3716 cord blood units in the CBS’ cord blood bank (CBB) have high-resolution HLA typing at five loci: 41.2% CAU, 25% API, 12.6% AFA, 2.9% HIS, and 26.5% OTH ethnicities. CBS’ CBB contains 202 redundant units (5.4%) comprising 78 HLA phenotypes, with varying rareness. Repeated phenotypes were from CAU donors (77%), multiple ethnicity (13%), API (9%), and AFA (1%). The rareness of the HLA phenotypes aligns with known haplotype frequencies, with AFA adult registrants and CBUs having the rarest phenotypes, while CAU donors have less-rare phenotypes than API, HIS, NAM, and OTH ethnicities.

**Conclusion:** Redundancy was greater in the SCR compared to the CBB at CBS and was the most common among CAU registrants and cord units. Greater ethnic diversity was observed in the cord blood bank, including many units with more rare HLA phenotypes compared to the SCR. Increased focus on recruiting non-Caucasian registrants and continued cord blood banking should lead to increased HLA match likelihoods for more patients searching for donors by reducing the overall redundancy. A subinventory of redundant donors and cord blood units could support new uses for donor-supported cellular therapies that do not require HLA matching.

**Abstract 14 (Poster):** A Portrait of Cord Blood Units Distributed for Transplantation from Canadian Blood Services’ Cord Blood Bank: First Analysis

Gaganvir Parmar ^1,2^, Meagan Green ^1^, Karen Mostert ^1^, Tiffany Lawless ^1^, Nicholas Dibdin ^1^, Jason Weiss ^1^, Kathy Ganz ^1^, Tanya Petraszko ^1,3^, Matthew D. Seftel ^1,3^, and David S. Allan ^1,4,5^

^1^  Stem Cells, Canadian Blood Services, Ottawa, ON, Canada
^2^  Faculty of Medicine, University of Toronto, Toronto, ON, Canada
^3^  Department of Medicine, Faculty of Medicine, University of British Columbia, Vancouver, BC, Canada
^4^  Department of Medicine and Biochemistry, Microbiology and Immunology, Faculty of Medicine, University of Ottawa, Ottawa, ON, Canada
^5^  Ottawa Hospital Research Institute, Ottawa, ON, Canada

**Background:** The Canadian Blood Services’ Cord Blood Bank (CBS’ CBB) was created to improve access to stem cell products for transplantation for patients across ethnic groups. An analysis of distributed units is needed to assess the effectiveness of the bank to meet the needs of patients from different ethnic groups.

**Methodology**: A descriptive analysis was performed on all the cord blood units distributed from the CBS’ CBB to 30 June 2022. 

**Results**: The distribution of the first 60 units based on CBS’ CBB inventory has been linear over time. A similar proportion of cord blood unit (CBU) recipients were pediatric or adult. More than half of the cord blood units (56.7%) were distributed to recipients outside of Canada, and CBUs were used to treat a broad range of hematological and immune disorders. A total of 43.3% of the distributed CBUs were of non-Caucasian ethnicity, and 18% were from donors self-reporting as multiethnic. The mean total nucleated cell counts and total CD34^+^ cell counts were 1.9 ± 0.1 × 10^9^ cells and 5.3 ± 0.5 × 10^6^ CD34^+^ cells, respectively. The CD34^+^ cells per kilogram (recipient weight) varied significantly between pediatric (ages 0–4), adolescent (ages 5–17), and adult recipients (ages 18 and older) (3.1 ± 0.5, 1.4 ± 0.5, and 0.9 ± 0.07 × 10^5^ CD34^+^ cells/kg, respectively). HLA matching was 6/6 (15%), 5/6 (47%), or 4/6 (38%).

**Conclusion**: The CBS’ CBB has facilitated the utilization of banked units for patients across a broad range of ages, geographic distributions, ethnicities, and diseases. Distributed units were well matched for HLA alleles and contained robust cell counts, reflecting a high-quality inventory with significant utility.

**Abstract 15 (Poster):** Modelling Unrelated Blood Stem Cell Donor Recruitment Using Simulated Registrant Cohorts: Assessment of HLA Matching Across Ethnicity Groups

John T. Blake ^1,2^, Gaganvir Parmar ^1^, Kathy Ganz ^1^, Matthew D. Seftel ^1,3^, and David S. Allan ^1,4,5^

^1^  Stem Cells, Canadian Blood Services, Ottawa, ON, Canada
^2^  Department of Industrial Engineering, Faculty of Engineering, Dalhousie University, Halifax, NS, Canada
^3^  Department of Medicine, Faculty of Medicine, University of British Columbia, Vancouver, BC, Canada
^4^  Department of Medicine, and Biochemistry, Microbiology and Immunology, Faculty of Medicine, University of Ottawa, Ottawa, ON, Canada
^5^  Ottawa Hospital Research Institute, Ottawa, ON, Canada

**Background:** HLA-matched unrelated donors are not available for some patients considered for allogeneic hematopoietic cell transplantation (HCT), particularly among certain ethnic groups. Simulated recruitment modelling can inform efforts to find new matches for more patients.

**Methodology**: Simulated recruits were generated by assigning a pair of donor HLA haplotypes from historical data files and matched against HLA data of patient searches in the Canadian Blood Services’ Stem Cell Registry. The recruitment cohorts reflected the proportion of five specific ethnic groups in the 2016 Canadian census data.

**Results:** Novel 8/8 HLA matches between simulated recruits and patients increased linearly with larger recruitment cohorts. The proportion of novel 8/8 HLA matches from Caucasian, Hispanic, and Native American/First Nations recruits was equal to or greater than their relative proportions in the recruited cohort (match: recruit ratio (MRR) ≥ 1). In contrast, African American and Asian and Pacific Islander recruits represented a smaller proportion of novel matches relative to their percentages of the recruited cohort (MRR < 1). The proportion of novel 7/8 HLA matches from each ethnic group was approximately the same as their proportion in the recruited cohort (MRR = 1), and high rates of 7/8 HLA-matching already exist within CBS’ registry for all the ethnic groups.

**Conclusion:** Continued large recruitment cohorts are needed to add new 8/8 HLA matches to registry inventories. Likelihoods of novel HLA matches varied across ethnic groups, reflecting varied HLA haplotype frequencies across groups. Simulated cohort modelling can inform recruitment strategies that will generate new donor options for patients.

**Abstract 16 (Poster):** Second-Line Management of Patients (pts) with Relapsed or Refractory Diffuse Large B-Cell Lymphoma (R/R DLBCL): Canadian Real-world Data

Isabelle Fleury ^1^, Theresa Amoloja ^2^, Zhenyi Xue ^2^, Caroline Koch ^3^, Eva E. Waltl ^4^, and Anthea Peters ^5^

^1^  Hôpital Maisonneuve-Rosemont, University of Montreal, Montreal, QC, Canada
^2^  Incyte Corporation, Wilmington, DE, United States of America
^3^  Incyte Biosciences Canada, Pointe-Claire, QC, Canada
^4^  MorphoSys AG, Planegg, Germany
^5^  Department of Oncology, University of Alberta, Edmonton, AB, Canada

**Background:** Approximately 35–40% of pts with DLBCL have R/R disease after first-line (1L) therapy. The current Canadian salvage strategy relies on eligibility for high-dose chemotherapy and autologous stem cell transplant (ASCT). Outcomes of pts ineligible for ASCT are poor. Understanding clinical characteristics of pts not eligible for ASCT is important to optimize their treatment options. Real-world data on the management of pts with R/R DLBCL are very limited in Canada. RE-MIND2 (NCT04697160) was designed to capture pt-level data for pts with R/R DLBCL to match the L-MIND study (NCT02399085) of tafasitamab + lenalidomide.

**Purpose:** To conduct a descriptive post hoc analysis of Canadian pts with R/R DLBCL enrolled in RE-MIND2 to better understand real-world pt characteristics and management after 1L.

**Methodology:** Two Canadian academic centres (Cross Cancer Institute, AB and Hôpital Maisonneuve-Rosemont, QC) participated in RE-MIND2. Data were retrospectively collected on pts diagnosed with DLBCL between 2010 and 2020. Eligible pts were ≥18 years with histologically confirmed DLBCL, who had received ≥2 systemic therapies (including ≥1 anti-CD20 therapy) administered according to NCCN/ESMO guidelines. Descriptive statistics were used in the analysis.

**Results:** Data from 109 Canadian pts with R/R DLBCL who met RE-MIND2 study criteria were included. Seventy-two out of 97 pts (74.2%) enrolled in 2L were considered as being eligible to receive ASCT. Table 1 summarizes the demographics and disease characteristics for pts who did or did not receive ASCT for 2L therapy. The median ages at the beginning of 2L were 55.5 y and 67.0 y for those who did and did not receive ASCT, respectively; the majority of pts were male in both groups (33 (75.0%) and 35 (66.0%)). Overall, 48 pts (44.0%) received ASCT (2L, 91.7%; 3L, 6.3%; 4L, 2.1%) and 15 pts (13.8%) received CAR T-cell therapy (2L, 0%; 3L, 66.7%; 4L, 33.3%). Of the 72 pts eligible for ASCT, 29 (~40%) did not proceed to transplantation. Chemorefractoriness (9 (37.5%)) and advanced age (10 (41.7%)) were the main reasons for ASCT ineligibility (Table 2). The most common prescribed 2L therapy for pts not receiving a transplant was gemcitabine, dexamethasone, and cisplatin ± rituximab (32%), followed by gemcitabine/oxaliplatin ± rituximab (5.2%) or bendamustine (5.2%). Pts who received conventional salvage chemotherapy in 3L and 4L had short median times to the next treatment (3.8 and 3.4 months, respectively).

**Conclusion:** Treatment patterns in Canadian pts with R/R DLBCL demonstrate that platinum-based treatment followed by ASCT is the standard-of-care 2L therapy for eligible pts. Chemorefractoriness and advanced age are the main reasons for ASCT ineligibility. Following conventional chemotherapy, pts ineligible for ASCT have poor outcomes. Alternative more efficacious salvage therapies are warranted to improve pt outcomes. Further DLBCL Canadian analyses are required to confirm these findings with available novel emergent therapeutic options.

**Abstract 17 (Poster):** Enteral Nutrition Leads to Less-Severe Gastrointestinal Mucositis and Fewer Days of Nutritional Support as Compared to Parenteral Nutrition Among Autologous Transplant Patients in a Randomized Study

Heather Resvick ^1,2^, Brenda Hartman ^1,3^, Cheryl Sigfrid ^2^, Victoria Friscioni ^3^, Janet Madill ^1,3^, Ellasha Cruikshank ^3^, Maisam Abouzeenni ^2^, Adrienne Fulford ^2,4^, Anargyros Xenocosta *^2^*^,4,5^, Robert Arntfield ^2,6^, and Uday Deotare *^2^*^,4,5,7^

^1^  Faculty of Health and Rehabilitation Sciences, Western University, London, ON, Canada
^2^  London Health Sciences Centre, London, ON, Canada
^3^  School of Food and Nutrition, Brescia University College, London, ON, Canada
^4^  Blood and Marrow Transplant Program, London Health Sciences Centre, London, ON, Canada
^5^  Division of Hematology, Department of Medicine, University of Western Ontario, London, ON, Canada
^6^  Division of Critical Care, Department of Medicine, University of Western Ontario, London, ON, Canada
^7^  The Centre for Quality, Innovation, and Safety, Schulich Medicine & Dentistry, University of Western Ontario, London, ON, Canada

**Background:** Inadequate oral intake and treatment-induced malnutrition often result from side effects, like mucositis and GI disturbances, associated with autologous hematopoietic stem cell transplants (AHSCTs). The American Society of Enteral and Parenteral Nutrition (ASPEN) recommends nutritional support via enteral (EN) or parenteral (PN) nutrition in this patient population. PN is commonly used to address these side effects as the standard of care at our centre, secondary to central line catheter accessibility.

**Purpose:** To examine differences in transplant-related, biochemical, and nutrition-related outcomes in AHSCT patients randomized to enteral vs. parenteral nutritional support groups.

**Methodology:** This (pilot) prospective randomized study was conducted at London Health Sciences Centre in London, ON, Canada. AHSCT patients (*n* = 37, 54% female) were recruited between 2019 and 2022 and randomized to receive either EN (46%) or PN (54%) support. Nutritional supports were initiated when oral intake fell below 80% of the estimated needs for energy and protein. Patients were assessed at three time points: baseline (at admission), day 15, and day 30 post transplant. Assessments included anthropometric (BMI), biochemical (albumin, pre-albumin, CRP, and blood glucose), nutritional (standardized phase angle (SPhA), bioelectrical impedance analysis (BIA), quadricep muscle layer thickness (QMLT), and handgrip strength (HGS)), and dietary measures (3-day food record).

**Results:** No significant differences were found between EN and PN groups for the length of the hospital stay or biochemical measures. The mean length of the nutritional support was 6.60 ± 3.40 and 10.63 ± 6.02 days for EN vs. PN, respectively. Over the study period, there were fewer incidences of gastrointestinal mucositis of any grade and fewer incidences of grade 3 in the EN group compared to the PN group, 35.5% vs. 55%, respectively. There were no significant differences between the groups at the baseline, day 15, or day 30 for SPhA.

**Conclusion:** Fewer cases overall and less-severe cases of GI mucositis were found in the enteral nutritional support group. Patients randomized to enteral vs. parenteral nutritional support did not have a longer length of stay in the hospital, nor were there any significant differences in laboratory measures and SPhA between the two groups. Our results support the use of enteral nutrition vs. parenteral to mitigate treatment-induced malnutrition in AHSCT patients. A multicentre study is needed to support these findings further. More data will be available and presented at the CTTC conference in June 2023.

**Abstract 18 (Poster):** The abstract was withdrawn. The authors were unable to attend and present.

**Abstract 19 (Poster):** Real-World Experience of the Standard-of-Care (SOC) CAR T-cell Therapy Program at Princess Margaret Cancer Centre, Toronto, ON, Canada

Sita Bhella, Carmel Waldron, Andrew Winter, Rachel Aitken, Aatikah Azeezmohideen, Kai Iwano, Katrina Hueniken, Anca Prica, Danielle Rodin, Rhida Bautista, Eshrak Al-Shaibani, Michael Crump, Vishal Kukreti, John Kuruvilla, Robert Kridel, Rodger Tiedemann, Chloe Yang, Wilson Lam, Arjun Law, Ivan Pasic, Sam Saibil, David Hodgson, Richard Tsang, and Christine Chen

Princess Margaret Cancer Centre, Toronto, ON, Canada

**Background:** Princess Margaret (PM) is the referral centre for CD19 CAR T-cell therapy (CART) for a large local population and a national referral centre. A better understanding of early CART failure may inform selection criteria.

**Purpose:** We evaluated the outcomes of all the patients referred for CART and explored risk factors for early failure, as defined by the failure to receive cells, death within 100 days of infusion, or progressive disease (PD) by the first-response assessment.

**Methodology:** This is a single-centre retrospective review of all the patients with RR-DLBCL referred for CART at PM from June 2020 to August 2022. Eligible patients had RR-DLBCL after 2+ prior systemic therapies and had eGFR > 45 mL/min/1.73 m^2^, LVEF > 40%, pulse oxygenation > 91% in room air, a performance status (KPS) of > 70%, and no active CNS disease. Tisa-cel was approved before axi-cel in Canada.

**Results:** A total of 148 patients (pts) were referred for CART during the study period. Of these, 107 (72%) received CART (tisa-cel 41 and axi-cel 66) (Table 1). The median time from acceptance for CART to infusion was 46 (19,100) days, 50 days (19,100), and 44 days (28–89) for all the pts, tisa-cel, and axi-cel, respectively. The median time from apheresis to infusion was 37 days (27,107), 43 days (35,107), and 34 days (27,69) for all the pts, tisa-cel, and axi-cel, respectively. The CRS of any grade occurred in 93/107 pts, with one grade 3+. Twenty-seven of the 107 pts developed ICANS, three (3%) developed grade 3+ ICANS. Eight required ICU support (8%) (2 ICANS, 2 CRS, and 4 Other) The response at 100 days was assessed in 107 pts: 34 achieved CR/CMR (32%), 12 achieved PR (11%), 34 (32%) experienced progression, and two (2%) died before 100 days; the 100-day status was unknown/pending in 25 (23%) patients. The median OS from consultation was 12.91 months for the cohort and 25.79 months among patients receiving CART. The median survival for patients not infused with CART was 2.46 months (Figure 1). The median follow-up times from infusion were 6.93 months, 20.76 months, and 5.75 months for all infused pts, tisa-cel, and axi-cel, respectively. The median PFS was 8.26 months, 1.91 months, and not-yet reached (NYR) for all the infused pts, tisa-cel, and axi-cel (*p* = 0.02). The median OS was 24.21 months, 12.32 months, and NYR for all the infused pts, tisa-cel, and axi-cel (*p* = 0.20), respectively. A univariate analysis of the predictors for early failure was conducted. Those with an unknown status at day 100 were excluded. A total of 123 pts were included, and 75 (61%) pts met the definition of early failure. The examined variables included the stage, ECOG score, presence of bulky disease (>7 cm), relapsed/refractory disease, lymphoma subtype, cell of origin, presence of double-hit or triple-hit lymphoma, and failure to undergo ASCT. Only the failure to undergo ASCT was significant (*p* = 0.002).

**Conclusion:** We present the early experience with SOC CART for RR-DLBCL from a large tertiary Canadian centre. Our results are similar to other RWEs. Limited RW cohorts have provided ITT results. A large proportion of referred patients experienced early failure, with 27.7% not receiving CART. Improved pt selection criteria are worthy of further investigation.

**Figure 1:**
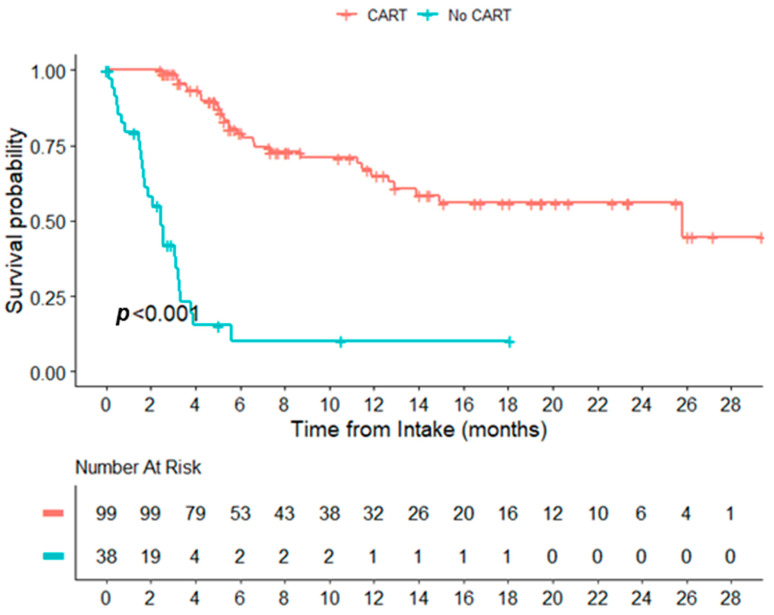
Overall survival from intake date, CAR T-cell therapy vs. no CAR T-cell therapy.

**Abstract 20 (Poster)**: Prognostic Impact of Donor Chimerism after Allogeneic Stem Cell Transplants (**Award Recipient**—Clinical Trials/Observations)

Michael Radford ^1^, Nida Usmani ^2^, Elaine Jin ^3^, Rohail Badami ^4^, Elizabeth McCready ^5,6^, Daria Grafodatskaya ^5,6^, Kylie Lepic ^1^, Alejandro Garcia-Horton ^1^, and Tobias Berg ^1,7,8^

^1^  Department of Oncology, McMaster University, Hamilton, ON, Canada
^2^  Division of Pediatric Hematology–Oncology, Charles Bruneau Cancer Centre, CHU Sainte-Justine, Montreal, QC, Canada
^3^  Department of Medicine, McMaster University, Hamilton, ON, Canada
^4^  Department of Interdisciplinary Science, McMaster University, Hamilton, ON, Canada
^5^  Department of Pathology and Molecular Medicine, McMaster University, Hamilton, ON, Canada
^6^  Hamilton Regional Laboratory Medicine Program, Hamilton Health Sciences, Hamilton, ON, Canada
^7^  Centre for Discovery in Cancer Research, McMaster University, Hamilton, ON, Canada
^8^  Escarpment Cancer Research Institute, Hamilton Health Sciences, Hamilton, ON, Canada

**Background**: Allogeneic stem cell transplant (SCT) is one of the few currently available therapies to cure acute myeloid leukemia (AML) and myelodysplastic syndrome (MDS). Positive outcomes are dependent on the engraftment of donor cells to provide hematopoiesis as well as a graft-versus-tumor effect. The measurement of the whole-blood (WB) and T-cell (TC) chimerism at routine intervals post SCT allows for the monitoring of the engraftment, particularly in settings where more sophisticated measures are not available or feasible. The development of mixed-donor chimerism (MC) may indicate impending graft failure or disease relapse. The identification of MC early post transplant can assist with prognostication as well as guide potential interventions, which could positively impact the trajectory of the disease course.

**Purpose**: The primary purpose was to evaluate whether MC is associated with poorer overall survival (OS).

**Methodology**: Adults with AML or MDS who received an SCT between 1 January 2016 and 1 February 2022 and had at least one donor chimerism measured on day 30 (D30), day 60 (D60), or day 90 (D90) at Juravinski Hospital (Hamilton, ON, Canada) were retrospectively analyzed. Complete chimerism (CC) was defined as ≥95%, whereas MC was defined as ≤95%. All the chimerism measurements were measured using a PowerPlex® 16 system (Promega) (Hamilton Regional Laboratory Medicine Program, Hamilton, ON). OS was estimated using the Kaplan–Meier method. Log-rank tests were used to compare survival curves.

**Results**: A total of 143 patients with a median age of 63 years (19–76) were transplanted for AML (*n* = 105), MDS/AML (*n* = 20), and MDS (*n* = 18). The relative proportion of participants with WB CC was similar between D30 (78%) and D90 (77%), with a slight reduction at D60 (74%) in MC. The relative proportion of participants with TC CC increased between D30 and D90, while TC MC decreased. The numbers of patients who improved from MC at D30 to CC at D90 in WB and TC were five and 14, respectively. The median OS was not reached for WB and TC CC at all the measurement time points and was significantly longer for patients at all the time points compared to patients with MC. The D30 WB and TC median OS were 9.1 months and 19 months, respectively. Overall, 55 of 143 (38.5%) patients in the cohort died. OS was significantly different between CC and MC for WB and TC at all three time points (*p* < 0.025). D30 WB MC was found to be a strong early prognostic indicator (hazard ratio: 2.67) as well as D30 TC MC (hazard ratio: 2.27). Cellular therapy interventions associated with MC included donor lymphocyte infusion (DLI) (*n* = 7) and a second transplant (6). A total of 6 of 6 (100%) second-transplant patients died, while 6 of 7 (86%) DLI patients were alive. See Table 1 and Table 2 and Figure 1.

**Conclusion**: Mixed-donor chimerism remains a strong prognostic indicator at all stages in first 90 days post SCT. This provides evidence to support further investigation into the beneficial impact of earlier interventions, such as DLI, or novel interventions post SCT.

**Figure 1:**
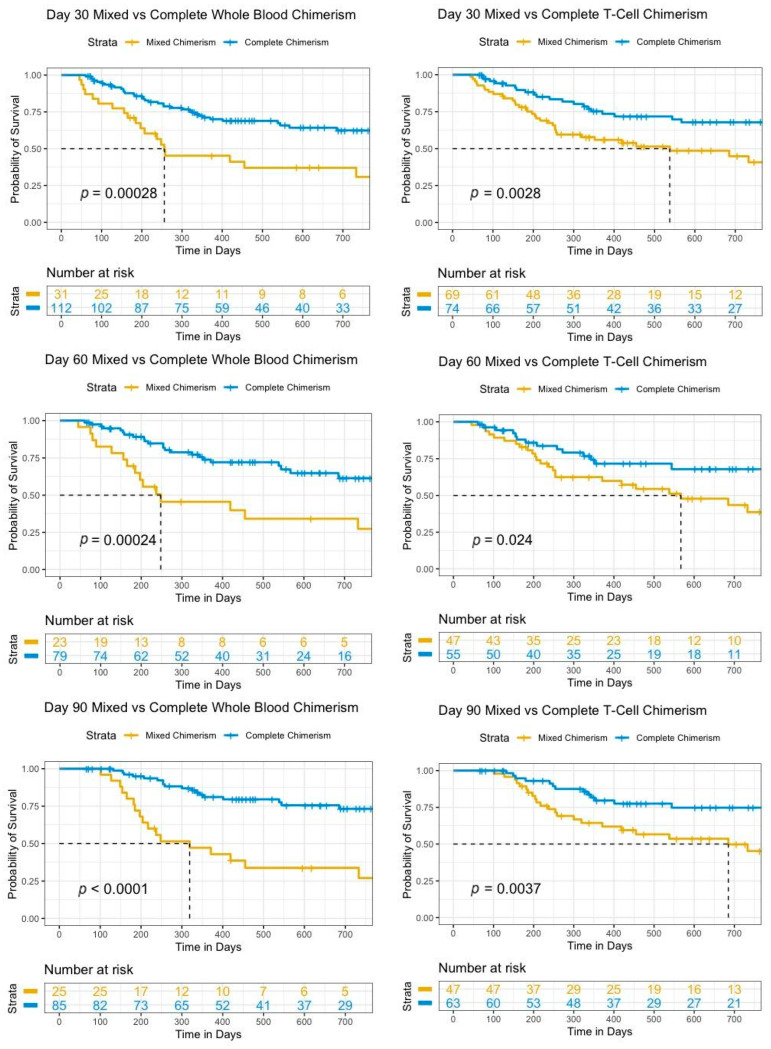
Survival curves for mixed versus complete whole-blood or T-cell chimerisms at day 30, day 60, and day 90.

**Abstract 21 (Poster):** Flow Cytometry Verification and Qualification Studies: A Stem-Cell-Manufacturing Approach

Kelly Murphy ^1^, Meri Antille ^1^, Christine Welch ^1^ Nicholas Dibdin ^1^, Kathy Ganz ^1^, David Allan ^1,2^, Matthew D. Seftel ^1,3^, and Jelena L. Holovati ^1,4^

^1^  Stem Cells, Canadian Blood Services, Edmonton, AB, Canada
^2^  Department of Medicine and Biochemistry, Microbiology and Immunology, University of Ottawa, Ottawa, ON, Canada
^3^  Department of Medicine, University of British Columbia, Vancouver, BC, Canada
^4^  Department of Laboratory Medicine and Pathology, University of Alberta, Edmonton, AB, Canada

**Background:** Flow cytometers are key analyzers in stem cell biomanufacturing, performing both CD34+ enumeration and phenotypic characterization of cell therapy products essential for identity, dosage, viability, and potency evaluation of cell therapy products. This system analyzes individual cells as they are hydrodynamically focused and flow past a laser. Photodetectors collect electronic and optical data from individual cells as they hydrodynamically cross the laser beam to allow for the phenotypic classification of cells based on multiple parameters detected during acquisition. The Canadian Blood Services’ (CBS’) Stem Cell team currently uses a Beckman Coulter FC500 flow cytometer with a locked StemCXP algorithm for the automated gating of stem cell samples.

**Purpose:** The aim of this study is to present a structured approach to flow cytometry verification studies through the implementation of the new Beckman Coulter Navios EX flow cytometry system in the CBS’ stem cell biomanufacturing environment.

**Methodology:** Verification studies included examining the accuracy and precision of the Beckman Coulter Navios EX flow cytometers in our environment, including the Stem-kit, the Cyto-Stat triChrome CD45-FITC/CD-19-RD1/CD3-PC5, and the Cyto-Stat tetraChrome CD45-FITC/CD4-RD1/CD8-ECD/CD3-PC5, as lab-developed tests. In addition, inter- and intra-instrument parallel studies coupled with a linear regression evaluation have been performed to correlate FC500 to the Navios EX instruments on a variety of stem cell sample types, including mobilized peripheral whole blood, hematopoietic progenitor apheresis cell products, cord blood, bone marrow, and non-mobilized mononuclear cell products.

**Results:** The installation and operational qualification verification determined that the instruments have been installed according to the manufacturer’s recommendations and configured to operate as required by the specifications. Parallel CD34 enumeration scattergrams of different sample types demonstrated strong linear correlations with the following acceptable R^2^ values: TNC% viability, 0.9961; CD45% viability, 0.9989; CD34% viability, 0.8327; leukocyte count (×10^3^/μL), 0.9685; viable CD34/μL, 0.995; total CD34/μL, 0.9835. Similarly, phenotyping scatterplots show a close fit of the linear regression trendline with the parallel testing data (R^2^ values range from 0.9818 to 0.9987). A comparison to the reference value obtained with StemCXP was used to optimize the lab-developed tests, in particular, the shape of the regions, as in the absence of the StemCXP algorithm, the regions are not auto adjusted to the shape of the target population.

**Conclusion:** This multipronged verification and qualification approach demonstrates the acceptable performance of the Beckman Coulter Navios EX flow cytometry system for quantitative and qualitative analyses of different types of stem cell samples, supporting the upcoming implementation of these analyzers in our stem-cell-manufacturing laboratories.

**Abstract 22 (Poster):** “Never Say Never” for Transplants in Aplastic Anemia

Zoe Evans ^1,2^, James Smith ^1^, Katie McNamara ^1^, Jonas Mattsson ^1,3,4^, and Rajat Kumar ^1,3^

^1^  Hans Messner Allogeneic Blood and Marrow Transplant Program, Princess Margaret Cancer Centre, Toronto, ON, Canada
^2^  Lawrence S. Bloomberg Faculty of Nursing, University of Toronto, Toronto, ON, Canada
^3^  Department of Medicine, University of Toronto, Toronto, ON, Canada
^4^  Gloria and Seymour Epstein Chair in Cell Therapy and Transplantation, Toronto General and Western Hospital Foundation, University Health Network, Toronto, ON, Canada

**Background:** In severe aplastic anemia (SAA), allogeneic blood and marrow transplantation (BMT) is a curative treatment option for eligible patients. The BMT team is often faced with a clinical conundrum when evaluating patients with concurrent severe infections and poor performance statuses who are referred for transplants. The following case study aims to highlight a successful BMT in a patient with multiple severe infections through a creative multidisciplinary approach.

**Purpose:** To highlight a successful peripheral blood stem cell transplant in a patient with SAA who was toxic, with multiple refractory concurrent infections, and bed-ridden, with a large nonhealing perianal ulcer restricting him to a prone position.

**Case:** A 33-year-old male with a diagnosis of SAA versus hypoplastic MDS was referred for a transplant. He had been hospitalized for the previous 4 months because of multiple infections refractory to the treatment. He had a large necrotizing peri-anal and sacral wound, measuring 11 cm × 9.5 cm, secondary to a hemorrhoidectomy performed while he was neutropenic. He was febrile, toxic, bed-ridden, and on daptomycin and meropenem for persistent multiorganism bacteremia with *Enterococcus gallinarum* and *Stenotrophomonas maltophilia*. He was also on amphotericin and isavuconazole therapy for a possible invasive fungal pneumonia based on radiological findings. His KPS was 20% (hospitalized and bed-ridden), and he was only able to lie in the prone position. As a lifesaving measure, he was considered for transplant. Because of the diagnostic overlap between SAA and hypoplastic MDS, the chosen conditioning regimen was fludarabine and busulfan (FB2), and GvHD prophylaxis was with anti-thymoglobulin, methotrexate, and cyclosporine. Total body irradiation (TBI) was omitted because of the inability of the patient to lie in the supine position. He underwent a matched-sibling donor transplant using peripheral blood stem cells (PBSCs), with a CD34 cell dose of 8.17 × 10^6^ cells/kg of bodyweight. The graft source was PBSCs rather than bone marrow to facilitate rapid engraftment. The infected wound was 11 × 9.5 cm, with 75% slough (images will be shown). The measures to manage the wound were (a) a barrier wipe to protect the peri-wound and Hydrofera Blue classic applied to the wound bed, (b) a diluted solution of 50% antibacterial MEDIHONEY, (c) hyperbaric oxygen before and after the transplant, (d) broad-spectrum antimicrobials, (e) total parental nutrition, (f) pain management, and (g) aggressive physiotherapy. The outcome was successful, with neutrophil engraftment on day +14 and platelet engraftment on day +31. On day +86, he was transferred to an inpatient rehabilitation centre and subsequently discharged home on day +93. All the antimicrobials were stopped by day +120. The wound healed, and he remains well and independently ambulant, with no graft-versus-host disease.

**Conclusion**: A multidisciplinary team approach can be lifesaving in patients with SAA who may be considered as being ineligible for BMT because of their active co-morbidities.

**Abstract 23 (Poster):** Medication Utilization for the Management of Toxicities Associated with CAR T-Cell Therapy: The Nova Scotia Experience

Laura V. Minard ^1^, Christina Fraga ^2^, Amye M. Harrigan ^2^, Kathy Walker ^1^, Jim Godin ^1^, Katrina Killen ^1^, Deanna Bowley ^1^, Jennifer Sweetapple ^1^, Megan Rolle ^1^, Josee Rioux ^3^, Erica C. MacLean ^1^, Kelsey Mann ^1^, Darrell White ^2^, and Mahmoud Elsawy ^2^

^1^  Pharmacy Department, Nova Scotia Health, Halifax, NS, Canada
^2^  Division of Hematology and Hematologic Oncology, QEII Health Sciences Centre, Halifax, NS, Canada
^3^  Cancer Care Program, Nova Scotia Health, Halifax, NS, Canada

**Background:** Chimeric-antigen-receptor (CAR) T-cell therapy recently became available in Nova Scotia for adults with relapsed or refractory large B-cell lymphomas who have received two or more previous lines of therapy. Given the common and expected toxicities associated with CAR T-cell therapy, it is important to characterize these toxicities and understand how they are managed in the early phases of the Nova Scotia program.

**Purpose:** To characterize toxicities associated with CAR T-cell therapy and describe medication utilization for their management during the first year of the Nova Scotia program.

**Methodology:** A chart review of patients who received CAR T-cell therapy during hospital admission in Nova Scotia between 4 April 2022 and 9 January 2023 was performed. Patient demographics and treatment-related information were collected. The presence or absence of cytokine release syndrome (CRS) and immune-effector-cell-associated neurotoxicity syndrome (ICANS) was determined, including grade, onset, and duration. Details on the utilization of medications to manage toxicities were collected.

**Results:** Ten patients received CAR T-cell therapy in Nova Scotia since the program launched. All the patients received lymphodepleting chemotherapy with fludarabine/cyclophosphamide, followed by CAR T-cell therapy with axicabtagene ciloleucel. The median patient age was 60.5 years (range: 23–75), and 70% of the patients were female. Forty percent of the patients were diagnosed with diffuse large B-cell lymphoma (DLBCL), 40% with DLBCL arising from follicular lymphoma, and 20% with high-grade B-cell lymphoma. The patients had received a median of two previous lines of therapy. Sixty percent of the patients received bridging therapy prior to CAR T-cell infusion. The median length of the hospital admission was 18 days, and 70% of the patients required admission to the ICU. All the patients experienced CRS (20% ≥ grade 3), with a median onset of 1 day and a median duration of 6 days (Table 1). Nine patients received tocilizumab, and two patients required vasopressor support (Table 2). Eight patients experienced ICANS (50% ≥ grade 3), with a median onset of 5 days and a median duration of 10 days. Eight patients received varying doses of corticosteroids. One patient received anakinra for steroid-refractory ICANS. All the patients were treated with broad-spectrum antibiotics in response to fever and received prophylaxis for *Pneumocystis jirovecii* pneumonia upon cell count recovery. All the patients received valacyclovir and fluconazole prophylaxis. In five cases, patients were changed to caspofungin after receiving high doses of steroids for three or more days.

**Conclusion:** Most Nova Scotia patients treated with CAR T-cell therapy experienced CRS and ICANS and were successfully managed with tocilizumab and/or corticosteroids, with a full resolution of the symptoms. It will be important to continue to closely monitor these toxicities and investigate the implementation of mitigation strategies to improve safety as the program expands.

**Abstract 24 (Poster):** Increasing the Rates of Comprehensive Chronic Graft-vs-Host Disease (cGvHD) Screening in Pediatric Blood and Marrow Transplant (BMT) Patients Using Electronic Screening Questionnaires: A Quality Improvement Project (**Award Recipient**—Pharmacy, Nursing, and Other Transplant Support)

Juliana Park Roden ^1^, Alison Vavra ^1^, Cassandra Managh ^1^, Jacob Rozmus ^1^, Jennifer Disabato ^2^, and Amanda Li ^1^

^1^  British Columbia Children’s Hospital (BCCH), Vancouver, BC, Canada
^2^  Children’s Hospital Colorado Anschutz Medical Campus, Aurora, CO, United States of America

**Background and Purpose:** Chronic graft-vs-host disease (cGvHD) is a significant complication of allogeneic blood and marrow transplant (BMT), affecting up to 70% of BMT recipients, and is the most common cause of non-relapse mortality (Baird et al., 2010). cGvHD can lead to inflammation and fibrosis, leaving patients with sequelae that are difficult to reverse. Comprehensive screening in BMT patients is crucial to identify emerging GvHD symptoms, initiate treatment early, and avoid progression to severe forms. At BCCH, a baseline audit of cGvHD-screening practices by BMT providers revealed that an average of four out of seven target organs were regularly screened for post BMT. Prior to this project, BMT providers did not have a standardized cGvHD-screening method. The primary aim was to increase the mean number of target organs screened for cGvHD by providers in post BMT patients.

**Methodology:** This quality improvement (QI) project, conducted within BCCH’s BMT program, used a modified version of Carpenter’s (2011) cGvHD-screening questionnaire that was e-mailed to patients via a secure webserver (REDcap). The responses were received and forwarded to the appropriate provider for interpretation prior to the clinic visit and then filed in the electronic health record.

**Results:** Patient questionnaires (*n* = 119) were completed. The number of organs screened by providers increased from a baseline of 2.71 to 6.75 organs post intervention. These data were analyzed using the Institute for Healthcare Improvement’s run chart rules. Accurate cGvHD grading in cGvHD patients increased from 32% pre-intervention to 66% post intervention, as analyzed with a chi-squared test (*p* = 0.002). Provider self-reported comfort and efficiency with cGvHD screening also improved post intervention.

**Conclusion:** The electronic questionnaires completed by the patients and the responses sent to the providers significantly improved the comprehensiveness of the cGvHD screening. Using this intervention, there is great potential to decrease non-relapse mortality and improve the quality of life for pediatric allogenic BMT recipients. BMT providers should consider using electronic methods to screen for GvHD symptoms and enhance communication with patients.

**References** **:**
Baird K, Cooke K, and Schultz KR. Chronic graft-versus-host disease (GvHD) in children. Pediatric Clinics of North America, 57 (1):297–322, 2010.
Carpenter PA. How I conduct a comprehensive chronic graft-vs-host disease assessment. Blood, 118 (10):2679–2687, 2011.

**Abstract 25 (Poster):** Dosing Strategies When Converting Cyclosporine from IV to PO Concomitantly with Starting Posaconazole Post Allogeneic Stem Cell Transplantation: Results of a Single-Centre, Retrospective Descriptive Cohort Study

Christopher Tse ^1^, Ian Pang ^1^, Kelly Guo ^1^, and Arjun Law ^2^

^1^  Department of Pharmacy, University Health Network, Toronto, ON, Canada
^2^  Hans Messner Allogeneic Blood and Marrow Transplant Program, Division of Medical Oncology and Hematology, Princess Margaret Cancer Centre, Toronto, ON, Canada

**Background and Purpose:** Cyclosporine A (CsA) is a calcineurin inhibitor used to prevent graft-versus-host disease post allogeneic stem cell transplant (Allo-SCT). These patients are also at risk of invasive fungal infections and require antifungal prophylaxis. Cyclosporine and posaconazole have a known drug interaction through CYP3A4 yet are often given concurrently. To the best of our knowledge, no studies have described the optimal CsA dose conversion when concurrently changing CsA intravenous (IV) to oral (PO) and IV echinocandins to PO posaconazole. Primary outcome: The proportion of the initial CsA trough levels within the therapeutic range was assessed after CsA IV:PO conversion and the initiation of PO posaconazole, and guiding principles were developed for CsA IV:PO conversion with the concurrent initiation of PO posaconazole. Secondary outcome: The effectiveness of our current CsA therapeutic drug monitoring was evaluated.

**Methodology:** A retrospective chart review was conducted for 200 patients who underwent Allo-SCT and had concurrent conversion of CsA IV:PO and IV echinocandins to PO posaconazole between 1 July 2019 and 1 November 2021. Cyclosporine trough levels were collected from the initiation of the CsA until discharge from the hospital. Patients were stratified based on the most recent CsA trough level prior to the CsA IV:PO conversion (subtherapeutic, within target range, or supratherapeutic).

**Results:** Fifty-nine percent of the patients had their first post-conversion CsA trough level within the target range (150–250 µg/L). The average CsA IV:PO conversion was 1:0.73. Patients who were supratherapeutic before the conversion were less successful than patients who were within the target range or subtherapeutic (55.9%, 63.6%, and 60%, respectively). The factors that correlated with less-successful CsA IV:PO conversion were obesity, patient receipt of matched donor stem cells or bone marrow stem cells, and if only one of PTCy and ATG was used in GvHD prophylaxis.

**Conclusion:** Our practice of the concurrent conversion of CsA IV:PO and IV echinocandins to PO posaconazole can be further optimized to ensure CsA levels are in the therapeutic range. Future studies can investigate which CsA IV:PO conversion leads to higher success rates.

**Abstract 26 (Oral):** Remote Monitoring of Patients After Allogeneic Stem Cell Transplantation: a Mobile Phone Application to Report Symptoms, Vital Signs, and Activities in Real Time (**Award Recipient**—Pharmacy, Nursing, and Other Transplant Support)

Armin Gerbitz ^1,2^, Eshrak al Shaibani ^1^, Andrew Quirke ^2^, Abir Abbas ^2^, and Rajshri Jayaraman ^2,3,4^

^1^  Princess Margaret Cancer Centre, Toronto, ON, Canada
^2^  Curetrax Inc., Toronto, ON, Canada
^3^  European School of Management and Technology, Berlin, Germany
^4^  Department of Economics, University of Toronto, Toronto, ON, Canada

**Background**: Some of the major obstacles for early inpatient discharge after allogeneic stem cell transplantation (aHSCT) are, aside from frailty and the required bloodwork, the high risk for infections and the need for monitoring graft-versus-host disease (GvHD). This requires frequent hospital visits, which are often a challenge, especially when travelling to hospitals imposes significant costs for the patient. Furthermore, structured data acquisition in the homecare setting is missing.

**Methodology**: We have developed a fully patient-centred, easy-to-operate mobile phone application for aHSCT patients that allows for treatment-specific symptom reporting. In combination with a wearable device (smart watch), a blood pressure monitor, and a thermometer, this application incorporates the online monitoring of all the major vital signs in almost-real time. The application currently captures more than 50 metrics relevant for aHSCT and provides a photo function to capture skin images in a patient-specific image library. Daily symptom reporting can be performed as often as necessary, and data from wearable devices are obtained at a very high frequency. The user interface for the physician dashboard was developed using the React JavaScript library (https://legacy.reactjs.org/, accessed on 1 May 2024), whereas the mobile application utilized React Native (https://reactnative.dev/, accessed on 1 May 2024) to enable Android and iOS applications. The collection, storage, and presentation of patient data via the physician dashboard is facilitated using various AWS technologies, such as Lambda, S3, and Timestream. Data can be stored according to federal laws and regulations of the country the software is operating in.

**Results**: Daily symptom reporting by the patient is easy, comprehensive, and can be completed within minutes. Data from wearable devices are obtained 24/7 at a high frequency. All the data are displayed on a dashboard to the corresponding physician with only a few minutes’ delay. The system captures vital signs (blood pressure, temperature, pulse, breathing rate, oxygen saturation, etc.) and GvHD-related symptoms, such as skin rashes and the frequency/volume of bowel movements. It provides information based on the patient’s physical activity, food intake, fluid intake, and sleeping patterns in a structured fashion. The physician’s dashboard displays all the transplant-relevant information, like the HLA match, conditioning regimen, type of immunosuppression, ABO incompatibility, and serologies of the patient and donor. It displays all the data longitudinally and allows physicians to observe trends and developments. It provides a medication plan that captures the daily dosage and duration of the treatment. Access to the dashboard is centre specific and secured by a dual verification process. All the data can be obtained in a structured fashion for research purposes by the participating institution.

**Conclusion**: This mobile phone application allows for the remote monitoring of patients after aHSCT and is developed in a modular fashion that can be easily adapted to specific requirements in other cell therapies, such as CAR CD19 or autologous SCT.

**Abstract 27 (Poster):** Collecting Products for CAR T-Cell Therapy—One Centre’s Experience with Estimating Collected Targets

Cheryl Page and Lindsay Chalmers
Hamilton Health Sciences, Hamilton, ON, Canada

**Background:** Chimeric-antigen-receptor (CAR) T-cell therapy treatment requires the initial autologous collection of T-cells, which will be genetically reprogrammed via a viral vector to bind to specific cell surface antigens on target cells (CCO, 2023). An initial step in the production of a CAR T-cell therapy product is collecting the target cells from the autologous donor by apheresis. Apheresis collection requires specially trained nursing staff. There is variation in the target cell types for various CAR T-cell products. Tools designed at one centre to support apheresis nurses with collections targeting CD3+ cells needed to be adapted to target mononuclear cell (MNC) counts. These tools were used to train apheresis nurses to estimate the final product volume to attain the target cell count using the interim complete blood count (CBC) on the product.

**Purpose:** There are minimal tools and literature to guide the apheresis nurse to obtain target products. At our centre, a formula has been used to predict target yields based on interim product CBC results. This method had been used previously to successfully predict collected volumes with products targeting a CD3+ cell count. For products targeting specific MNC counts, this process was revised.

**Methodology:** The apheresis nurse collects an interim CBC on the product halfway through the collection procedure. The MNC count at the interim is determined from the results. Using the product volume collected at the time of the sampling, the volume of the product required to meet the target count is calculated. Nursing staff use a formula specific to a product target based on MNC or CD3+ cell counts to guide them on how long to continue the procedure. All the apheresis nurses collecting CAR T-cells were experienced stem cell collection nurses who have completed training on the collection policy and completed shifts with a competent preceptor utilizing competency records to track their progress. The nurses experienced in CAR T-cell collection using the previous formula to estimate the product volume for a specific CD3+ target were trained on the new formula to target MNC cells.

**Results:** With the support of the interim sample results and formula to guide them, apheresis nurses were able to successfully reach target MNC cells in the final product in a similar fashion to products with a CD3+ target.

**Conclusion:** Many factors affect the final number of target cells in the product. Tools, such as this centre’s formulae to estimate the final product volume based on interim product CBC results, help to support the training of apheresis nurses in this area. This unit-based quality improvement project could form the basis of additional studies on product collection variables to attain target cell counts.

## Figures and Tables

**Table 1:** Gene expression of pediatric HSCT patients according to NK cell cluster. Cluster 0 (light blue colour) refers to cytotoxic CD56^dim^ NK cells; Cluster 1 (orange colour) includes classic CD56^bright^ NK_reg_ cells; Cluster 2 (yellow colour) are Type I interferon-responsive NK_reg_ cells.

cGvHD and No cGvHD (*n* = 7)			cGvHD (*n* = 3)			Cluster
Gene	Log_2_FC	*p* Value	Gene	Log_2_FC	*p* Value	
*FGFBP2*	1.23	<1 × 10^−300^	*TSC22D3*	0.809164	<1 × 10^−300^	0
*CCL5*	1.20	<1 × 10^−300^	*PRF1*	0.788909	3.1 × 10^−287^	0
*GZMK*	1.55	<1 × 10^−300^	*AREG*	1.546448	<1 × 10^−300^	1
*XCL1*	1.50	<1 × 10^−300^	*CCL5*	1.244255	4.4 × 10^−219^	1
*IFIT2*	2.88	<1 × 10^−300^	*GZMB*	1.04792557	9.71 × 10^−88^	2
*PMAIP1*	2.85	<1 × 10^−300^	*AREG*	1.01938356	6.97 × 10^−55^	2

**Table 1:** Summary of key metabolites elevated or depressed at cGvHD onset and critical statistical values showing significant differences between control and pathological groups.

Metabolite	Type of Metabolite	Effect Ratio	*p*-Value	AUC
α-Ketoglutarate	Organic acid	2.64	1.42 × 10^−6^	0.74
Kynurenine	Amino-acid related	1.42	3.93 × 10^−2^	0.59
Glutamic Acid	Organic acid	1.44	3.0 × 10^−2^	0.61
Glutamine	Amino acid	0.69	2.0 × 10^−3^	0.71
**C8**	Medium-chain fatty acid	1.31	2.0 × 10^−2^	0.62

**Table 1:** Baseline patient and disease characteristics for patients with relapsed/refractory large B-cell lymphoma treated with axicabtagene ciloleucel in Nova Scotia.

Baseline Characteristics		*N* (%)
Age		Median 60 years old
		(Range: 23–75 years)
Sex		
	Female	6 (66)
	Male	3 (33)
Diagnosis		
	DLBLC	4 (44)
	Transformed Lymphoma	3 (33)
	High-Grade DLBCL	2 (22)
Stage		
	Limited	3 (33)
	Advanced	6 (66)
ECOG		
	0	2 (22)
	1	7 (77)
Hematopoietic Cell Transplant Comorbidity Index		
	0	4 (44)
	1	1 (11)
	2	2 (22)
	3	2 (22)
Number of Previous Treatment Lines		
	2	6 (66)
	3	2 (22)
	4	1 (11)
Prior Autologous Stem Cell Transplant		1 (11)
Bridging Therapy Before CAR T-cell Therapy		
	Yes	6 (66)
	No	3 (33)
Type of Bridging Therapy Before CAR T-cell Therapy		
	Steroids	1 (11)
	Systemic Chemotherapy	3 (33)
	Radiation Therapy	2 (22)

**Table 1:** Patient characteristics in high-risk MM population.

Baseline Characteristics	Tandem ASCT *n* = 25 (%)
**Age at Diagnosis, Median (Range)**	59.2 (30–67)
**Male**	15 (60%)
**Lab Values at Diagnosis, Median (Range)**	
Hemoglobin (g/L)	104 (57–151)
Platelets (×10^9^/L)	195 (78–359)
Neutrophils (×10^9^/L)	3.9 (1.1–11.1)
Calcium (mmol/L)	2.29 (1.81–2.64)
Creatinine (mmol/L)	77 (56–692)
**ISS Stage**	
I	5 (20%)
II	5 (20%)
III	9 (36%)
Unknown	6 (24%)
**Immunoglobulin Subtype**	
IgG	9 (36%)
IgA	11 (44%)
IgD	0
IgM	0
Light Chain	4 (16%)
Non-secretory	1 (4%)
**High-risk Cytogenetics**	
T (4:14)	8 (32%)
t (14:16)	5 (20%)
Del17p	7 (28%)
1qamp	8 (32%)
1delp + 1qamp	5 (20%)
**Induction**	
CyBorD	25 (100%)
DRD (Transition)	1 (4%)
PAD/CVD (Transition)	1 (4%)
**Maintenance**	
Yes	19 (76%)
No	6 (24%)
**Type of Maintenance**	
Lenalidomide ± Dexamethasone	2 (8%)
Proteosome Inhibitor ± Dexamethasone	4 (16%)
Lenalidomide and Proteosome Inhibitor ± Dexamethasone	9 (36%)
**Response**	
Post Induction	CR-0
	VGPR-16
	PR-9
1–3 Months Post Tandem #1	CR-0
	VGPR-20
	PR-5
1–3 Months Post Tandem #2	CR-2
	VGPR-22
	PR-2
	Relapse-1
	Unknown
12 Months Post Tandem #2	CR-4
	VGPR-18
	PR-1
12 Months Post Maintenance	CR-5
	VGPR-9
	PR-0
**Relapse—total**	**11 (44%)**
1–3 Months Post Tandem #2—1	
Post Tandem #2 and Maintenance Start—9 except 1 patient relapsed on day 2 of #2 tandem	

**Table 1:** Comparison between collected patients who proceeded and did not proceed to CAR T-cell infusion. *p*-Values of 0.04 or less are presented in bold text.

	CAR T-Cell Infusion *n* = 68	No CAR T-Cell Infusion *n* = 17	*p*-Value
**Demographics**			
Median Age (Range) at Time of CAR T-Cell Therapy Referral	60.6 (22–81)	64 (50.8–73.9)	0.22
Gender, *n* (%)			1
Female	26 (38.2%)	7 (41.2%)	
Male	42 (61.8%)	10 (58.8%)	
Referring Centre, *n* (%)			0.52
Within Province	54 (79.4%)	12 (70.6%)	
Out of Province (OOP)	14 (20.6%)	5 (29.4%)	
Diagnosis, *n* (%)			0.096
DLBCL	38 (55.9%)	8 (47.1%)	
High-Grade Lymphoma	11 (16.2%)	8 (47.1%)	
Transformed Follicular Lymphoma	12 (17.6%)	1 (5.9%)	
Primary Mediastinal B-Cell Lymphoma (PMBCL)	5 (7.4%)	0	
Others	2 (2.9%)	0	
**Factors at Time of Relapse**			
IPI Score at Relapse, *n* (%)			0.17
0–2	29 (42.7%)	4 (23.5%)	
>2	39 (57.4%)	13 (76.5%)	
CNS IPI at Relapse, *n* (%)			0.28
Low	11 (16.4%)	2 (12.5%)	
Intermediate	44 (65.7%)	8 (50.0%)	
High	12 (17.9%)	7 (37.5%)	
High Serum LDH at Relapse, *n* (%)	55 (80.9%)	17 (100.0%)	0.06
Disease Stage at Relapse, *n* (%)			0.72
I–II	13 (19.1%)	2 (11.8%)	
III–IV	55 (80.9%)	15 (88.2%)	
Bulky Disease at Relapse, *n* (%)	35 (51.5%)	13 (76.5%)	0.099
Extra-nodal Disease at Relapse, *n* (%)	41 (60.3%)	9 (52.9%)	0.58
Prior CNS Disease, *n* (%)	0	2 (11.8%)	**0.04**
**Factors at Time of CAR T-Cell Therapy Referral**			
Active CNS Disease, *n* (%)	0	4 (23.5%)	**0.001**
Prior Lines of Therapy, Median (Range)	3 (2–5)	3 (1–4)	0.28
Disease Status at Referral for CAR T-Cell Therapy, *n* (%)			0.16
Relapsed	33 (48.5%)	5 (29.4%)	
Refractory	35 (51.5%)	12 (70.6%)	
Double- or Triple-Hit Lymphoma, *n* (%)	11 (16.2%)	8 (47.1%)	**0.006**
Karnofsky Performance Status (KPS), *n* (%)			0.11
≥80	53 (77.9%)	10 (58.8%)	
<80	15 (22.1%)	7 (41.2%)	
ECOG, *n* (%)			0.15
0–1	55 (80.9%)	11 (64.7%)	
≥2	13 (19.1%)	6 (35.3%)	
Hematopoietic Cell Transplant Comorbidity Index (HCT-CI), *n* (%)			0.21
0–1	48 (70.6%)	12 (70.6%)	
2–3	16 (23.5%)	2 (11.8%)	
>3	4 (5.9%)	3 (17.7%)	
**Factors at Time of Apheresis Cell Collection**			
Elevated Ferritin Level, *n* (%)	63 (92.7%)	11 (64.7%)	**0.0003**
Elevated Serum C-Reactive Protein, *n* (%)	34 (50%)	8 (47%)	**0.016**
Median Peripheral Blood Absolute Lymphocyte Count, 10^9^/L (Range)	0.9 (0.1–3.4)	0.5 (0.1–1.6)	**0.028**
Median Peripheral Blood CD3, Counts/UL (Range)	685.5 (16–3662)	308 (22–4916)	0.057
Bridging Therapy (After Apheresis), *n* (%)	53 (77.9%)	12 (75%)	0.75
**Timeline Metrics**			
Median Days from Salvage Treatment to Apheresis (Range)	19.5 (5–45)	19.5 (6–30)	0.77
Median Days from Initial Visit to Apheresis (Range)	12 (2–35)	13 (1–33)	0.88

**Table 1:** Patient demographics and baseline disease characteristics for patients who received or did not receive ASCT for second-line therapy.

Parameter (*n* = 97)	ASCT (*n* = 44)	No ASCT (*n* = 53)
Age at Index Date, y *		
Median (Range)	55.5 (27–72)	67.0 (26–86)
<65 y	35 (79.5)	22 (41.5)
≥65 y	9 (20.5)	31 (58.5)
Sex, *n* (%)		
Women	11 (25.0)	18 (34.0)
Men	33 (75.0)	35 (66.0)
IPI Score, *n* (%)		
0–2	12 (27.3)	7 (13.2)
3–5	5 (11.4)	14 (26.4)
Missing	27 (61.4)	32 (60.4)
Number of Extra-nodal Sites, *n* (%)		
0 or 1	16 (36.4)	25 (47.2)
≥2	13 (29.5)	9 (17.0)
Missing	15 (34.1)	19 (35.8)
Ann Arbor Disease Stage, *n* (%)		
I or II	6 (13.6)	5 (9.4)
III or IV	21 (47.7)	25 (47.2)
Missing	17 (38.6)	23 (43.4)
ECOG Performance Status, *n* (%)		
0 or 1	24 (54.5)	27 (50.9)
≥2	5 (11.4)	6 (11.3)
Missing	15 (34.1)	20 (37.7)
Prior CNS Involvement, *n* (%)		
Yes	2 (4.5)	4 (7.5)
No	42 (95.5)	49 (92.5)
Primary Refractoriness, *n* (%)		
Yes	24 (54.5)	31 (58.5)
No	20 (45.5)	22 (41.5)
Elevated LDH (>ULN), *n* (%)		
Yes	15 (34.1)	30 (56.6)
No	21 (47.7)	15 (28.3)
Missing	8 (18.2)	8 (15.1)

* Index date is the date recorded when treatment was initiated for second-line therapy. ASCT, autologous stem cell transplant; CNS, central nervous system; ECOG, Eastern Cooperative Oncology Group; IPI, International Prognostic Index; LDH, lactate dehydrogenase; ULN, upper limit of normal.

**Table 2:** Summary of ASCT eligibility at the start of second-line therapy and reasons for ASCT ineligibility.

Parameter	*n* = 97
Patients Eligible for ASCT, *n* (%) *	
Yes	72 (74.2) ^†^
No	24 (24.7)
Reason for ASCT Ineligibility, *n* (%)	
Chemorefractory ^‡^	9 (37.5)
Relapsed After a Prior ASCT	1 (4.2)
Comorbidities	2 (8.3)
Advanced age	10 (41.7)
Other	1 (4.2)
Missing	1 (4.2)

* ASCT eligibility status of one patient was unknown. ^†^ High number of ASCT-eligible patients might be associated with younger age of patients in the study cohort (who are more likely to be transplant eligible than older patients). ^‡^ Defined as progressive or stable disease as the best response to chemotherapy. ASCT, autologous stem cell transplant.

**Table 1:** Summary of all the data collected in the study. Numbers are followed by percentages in parentheses unless otherwise mentioned.

	All Referred Patients * n * = 148	CAR T * n * = 107	Tisa-Cel * n * = 41	Axi-Cel * n * = 66
** Age ** (Mean (SD))	59.9 (12.4)	59.1 (12.8)	62.1 (12.2)	57.2 (12.9)
** Sex **				
Female	56 (38)	42 (39)	18 (44)	24 (36)
Male	92 (62)	65 (61)	23 (56)	42 (64)
** ECOG at Intake **				
0–1	79 (79)	66 (81)	26 (79)	40 (83)
2+	21 (21)	15 (19)	7 (21)	8 (17)
Missing	48	26	8	18
** Stage **				
1	2 (3)	1 (2)	0 (0)	1 (3)
2	7 (9)	6 (11)	3 (12)	3 (9)
3	17 (22)	13 (23)	6 (25)	7 (21)
4	50 (66)	37 (65)	15 (62)	22 (67)
Missing	72	50	17	33
** Bulky Disease **				
≤7 cm	76 (56)	58 (55)	22 (56)	36 (55)
>7 cm	59 (44)	47 (45)	17 (44)	30 (45)
Missing	13	2	2	0
** Prior Auto Transplant **				
No	100 (68)	65 (61)	22 (54)	43 (65)
Yes	48 (32)	42 (39)	19 (46)	23 (35)
** History of CNS Disease **				
No	133 (96)	104 (99)	41 (100)	63 (98)
Yes	6 (4)	1 (1)	0 (0)	1 (2)
Missing	9	2	0	2
** Relapsed or Refractory **				
Refractory	81 (59)	59 (55)	19 (46)	40 (61)
Relapsed	57 (41)	48 (45)	22 (54)	26 (39)
Missing	10	0		
** Bridging **				
None	52 (36)	29 (27)	12 (29)	17 (26)
Chemotherapy Alone	22 (15)	16 (15)	6 (15)	10 (15)
RT	29 (20)	25 (23)	5 (12)	20 (30)
Steroids	12 (8)	10 (9)	4 (10)	6 (9)
Chemo + RT	1 (1)	1 (1)	1 (2)	0 (0)
Chemo + Steroids	6 (4)	6 (6)	3 (7)	3 (5)
RT + Steroids	19 (13)	15 (14)	5 (12)	10 (15)
Chemo RT Steroids	5 (3)	5 (5)	5 (12)	0 (0)
Missing	2	0	0	0

**Table 1:** Baseline patient characteristics.

Variable	Category	Number
Age (Years)	Median (Range)	63 years (19–76)
Gender—n (%)	Female	58 (41%)
	Male	85 (59%)
Gender Mismatch—n (%)	Yes	55 (38%)
	No	88 (62%)
Graft Source—n (%)	PBSC	142 (99%)
	BM	1 (1%)
Diagnosis—n (%)	AML	105 (73%)
	MDS/AML	20 (14%)
	MDS	18 (13%)
Karnofsky Performance Score	Median (IQR)	90 (10)
Disease Status—n (%)	1st CR	91 (64%)
	2nd CR	13 (9%)
	HI	14 (10%)
	NR/SD	14 (10%)
	PIF	4 (3%)
	CR	6 (4%)
	Not Reported	1 (1%)
Donor Source—n (%)	MMUD	1 (1%)
	MRD	26 (18%)
	MMRD	32 (22%)
	MUD	84 (59%)
Conditioning Intensity—n (%)	MAC	29 (20%)
	RIC	114 (80%)
Conditioning Regimen—n (%)	FLUBU2	55 (39%)
	Baltimore	32 (22%)
	FLUBU4	27 (19%)
	FluMel	7 (5%)
	FLUTBI	20 (14%)
	Kroger	2 (1%)
Immunosuppression—n (%)	Cyclosporine + MMF	83 (58%)
	Cyclosporine + MTX	12 (8%)
	Cyclosporine	7 (5%)
	Cyclosporine + Siro + MM	5 (3%)
	Tacrolimus + MMF	35 (25%)
	Tacrolimus + Siro	1 (1%)
GvHD Prophylaxis—n (%)	ATG	90 (63%)
	PTCy	32 (22%)
	ATG + PTCy	10 (7%)
	NA	11 (8%)
ELN 2022—n (%)	AML with Recurrent Genetic Abnormality	44 (35%)
	AML NOS	22 (18%)
	AML with MDS-Related Cytogenetic Abnormality	19 (15%)
	AML with MDS-Related Gene Mutation	13 (10)
	MDS/AML NOS	10 (8%)
	AML with Mutated TP53	7 (6%)
	MDS/AML with MDS-Related Gene Mutation	7 (6%)
	MDS/AML with MDS-Related Cytogenetic Abnormality	3 (2%)
ELN 2022 AML Disease Risk—n (%)	Adverse	55 (44%)
	Intermediate	43 (34%)
	Favourable	27 (22%)
Graft—Mean (SD)	CD34+ Count (×10^6^/kg Cells)	8.15 (4.37)
IPSS-R MDS Risk—n (%)	Low	3 (17%)
	Intermediate	6 (33%)
	High	5 (28%)
	Very High	3 (17%)

**Table 2:** Whole-blood and T-cell chimerism measurements at day 30, day 60, and day 90.

Measurement Day	Whole Blood *n* (%)		Measurement Day	T-Cell *n* (%)	
D30 (*n* = 143)	≥95% Donor	112 (78%)	D30 (*n* = 143)	≥95% Donor	74 (52%)
	<95% Donor	31 (22%)		<95% Donor	69 (48%)
D60 (*n* = 102)	≥95% Donor	79 (73%)	D60 (*n* = 102)	≥95% Donor	55 (54%)
	<95% Donor	23 (23%)		<95% Donor	47 (46%)
D90 (*n* = 110)	≥95% Donor	85 (77%)	D90 (*n* = 110)	≥95% Donor	63 (57%)
	<95% Donor	25 (23%)		<95% Donor	47 (43%)

**Table 1:** Toxicities associated with CAR T-cell therapy in Nova Scotia patients.

Toxicity		
CRS	Yes—*n* (%)	10 (100%)
	Grade ≥ 3—*n* (%)	2 (20%)
	Median Onset (Range)—Days	1 (0–3)
	Median Duration (Range)—Days	6 (1–7)
ICANS	Yes—*n* (%)	8 (80%)
	Grade ≥ 3—*n* (%)	5 (50%)
	Median Onset (Range)—Days	5 (1–9)
	Median Duration (Range)—Days ^a^	10 (1–30)
Presentation of CRS and/or ICANS	Concurrent—*n* (%)	7 (70%)
	Sequential—*n* (%)	1 (10%)
	CRS Only—*n* (%)	2 (20%)

^a^ Ongoing upon hospital discharge (*n* = 2); CRS, cytokine release syndrome; ICANS, immune-effector-cell-associated neurotoxicity syndrome.

**Table 2:** Drug therapies used to treat toxicities associated with CAR T-cell therapy in Nova Scotia patients.

Drug Therapy	
Tocilizumab—*n* (%)	9 (90%)
Mean Number of Doses Per Patient ^a^	2.3
Corticosteroids—*n* (%)	8 (80%)
ICANS—*n* (%) ^b,c^	4 (50%)
CRS—*n* (%) ^b,c^	2 (25%)
ICANS and CRS—*n* (%) ^b,c^	2 (25%)
Mean Cumulative Doses (Prednisone Equivalents)—mg ^b^	2728.8
Dose ≥ 6000 mg—*n* (%)	3 (37.5%)
Dose ≥ 1000 mg and < 6000 mg—*n* (%)	1 (12.5%)
Dose < 1000 mg—*n* (%)	4 (50%)
Median Duration (Range)—Days ^b,d^	6 (2–20)
Anakinra—*n* (%)	1 (10%)
Vasopressors ^e^—*n* (%)	2 (20%)
Broad-Spectrum Antibiotics ^f^—*n* (%)	10 (100%)
Median Duration (Range)—Days	12 (5.25–17)

Notes: ^a^ *n* = 9; ^b^ *n* = 8; ^c^ reason for prescribing; ^d^ total number of days on which corticosteroids were received; ^e^ norepinephrine; ^f^ piperacillin/tazobactam and meropenem; CRS, cytokine release syndrome; ICANS, immune-effector-cell-associated neurotoxicity syndrome.

